# Flow-Stabilized Microfluidic Sensing of Thermally Induced Changes in Red Blood Cell Aggregation During Closed-Loop Circulation

**DOI:** 10.3390/bios16100575

**Published:** 2026-10-12

**Authors:** Yang Jun Kang

**Affiliations:** Department of Mechanical Engineering, Chosun University, 10, Chosundae 1-gil, Dong-gu, Gwangju 61452, Republic of Korea; yjkang2011@chosun.ac.kr; Tel.: +82-62-230-7052; Fax: +82-62-230-7055

**Keywords:** closed-loop fluidic circuit, RBC aggregation, flow stabilizer, microfluidic chip, thermally induced RBC alterations, flow stabilization, hemorheology

## Abstract

Red blood cell (RBC) aggregation is a sensitive indicator of hemorheological alterations, but conventional methods generally rely on intermittent or end-point measurements. This study presents a flow-stabilized closed-loop microfluidic platform for continuous measurement of the RBC aggregation index (AI) during controlled circulation and thermal exposure. The system integrates a peristaltic pump, hydraulic flow stabilizer (FS), microfluidic aggregation sensor, and thermal mixer to suppress flow pulsation and RBC sedimentation. The FS increases the characteristic flow-response time by approximately 17.6-fold, markedly attenuating pump-induced fluctuations. Under stabilized conditions, flow rates in the main channel and test chamber remain highly linear with the imposed flow rate, while stable AI measurements are maintained for at least 30 min. The platform also resolves dextran-dependent changes in RBC aggregation during continuous circulation. During thermal exposure from 25 to 47 °C, main-channel image intensity (*I_mc_*) and AI remain nearly constant up to 43 °C, whereas exposure at ≥45 °C causes a progressive increase in *I*_mc_ and a marked decrease in AI. These results demonstrate continuous detection of thermally induced changes in RBC aggregation at a fixed measurement temperature of 25 °C without interrupting circulation and provide a versatile framework for investigating progressive changes in aggregation behavior under controlled and reproducible in vitro conditions.

## 1. Introduction

Red blood cells (RBCs) are highly deformable cells that play essential roles in oxygen delivery to peripheral tissues and carbon dioxide transport from tissues to the lungs [1]. Efficient gas transport requires RBCs to circulate smoothly through the microvasculature, where they frequently encounter vessels comparable to or smaller than their resting diameter. Consequently, the hemorheological properties of RBCs are critical for maintaining normal microcirculatory blood flow. Blood rheology is mainly governed by hematocrit, plasma viscosity, RBC deformability, and RBC aggregation. At low shear rates, typically below approximately 100 s^−1^, RBC aggregation becomes increasingly significant, thereby increasing apparent blood viscosity. As the shear rate increases, RBC aggregates progressively disperse, resulting in a decrease in blood viscosity and the characteristic shear-thinning behavior of blood [2,3,4]. RBC deformability is also essential for microcirculatory flow because individual RBCs must undergo substantial deformation to pass through narrow microvessels [2,5]. Therefore, alterations in RBC aggregation or deformability can modify flow resistance and impair microvascular perfusion [6,7,8,9,10]. These hemorheological properties are sensitive to various physical and biochemical stresses. Among these stressors, thermal exposure is particularly relevant because its effects on RBCs depend strongly on both temperature and exposure duration. As temperature or exposure time increases, thermal alterations in RBCs become progressively more pronounced and may eventually result in membrane disruption and hemolysis [11,12,13]. Importantly, heat-induced changes in RBC mechanical and rheological properties can develop before extensive hemolysis becomes evident [14,15,16]. Thus, hemolysis alone may not adequately reflect the early stage of thermal injury. Therefore, monitoring RBC hemorheological properties during thermal exposure may provide additional information on the onset and progression of thermal alterations before irreversible cell damage occurs.

Conventional assessment of thermally induced RBC alterations mainly relies on techniques that measure individual mechanical or hemorheological properties [17,18,19,20]. For example, membrane elasticity is evaluated using parallel-plate flow systems [14], RBC deformability is measured by filtration-based methods [15], and aggregation is assessed using light reflectometry or viscometry [16,21]. More severe thermal injury is typically evaluated by measuring hemoglobin release or hemolysis [11,12,17]. These previous studies show that higher temperatures and longer exposure times progressively alter RBC membrane mechanics, deformability, aggregation behavior, and eventually membrane integrity. However, many conventional approaches assess RBC properties after predefined thermal treatment or at selected experimental conditions [12,16,22,23,24]. Consequently, they mainly provide discrete information and are less suitable for continuously tracking the progressive hemorheological changes that occur in the same RBC population during continuous heat exposure.

Recent microfluidic techniques offer improved control of the flow environment and enable sensitive assessment of RBC deformability, aggregation, and blood viscosity under dynamic conditions [25,26,27,28,29,30,31]. Indeed, microfluidic capillary networks have been shown to detect progressive changes in RBC deformability more sensitively than conventional ektacytometry, while recent microfluidic rheometry has successfully detected hemorheological alterations in thermally exposed RBCs [32,33]. Shin et al. developed a pressure-driven microfluidic system that simultaneously measured shear-dependent RBC aggregation and blood viscosity and demonstrated its ability to characterize heated blood [3]. Zheng et al. subsequently used a microfluidic constriction with electrical detection to distinguish heated RBCs from untreated cells based on changes in deformability [34]. The combined effects of temperature and fluid flow have also been investigated under dynamic conditions. Foo et al. showed that RBC deformability changed while cells were heated from 37 to 42 °C under fluid flow [35], while Rodríguez-Villarreal et al. demonstrated that increasing the temperature from 23 to 50 °C markedly altered the cell-free area of RBC suspensions flowing through a microfluidic channel [36]. More recently, microfluidic rheometry has enabled simultaneous assessment of RBC aggregation and blood viscosity in thermally treated samples. These studies detected marked changes in aggregation, viscosity, and sedimentation following exposure to elevated temperatures, with the responses depending on both temperature and exposure duration [32,33]. Together, these findings demonstrate that microfluidic platforms provide sensitive functional measurements of thermally induced alterations in RBCs.

Overall, previous studies show that microfluidic measurements of RBC deformability, aggregation, and blood viscosity can sensitively detect thermal stress-induced changes. However, most studies employ a sequential exposure–analysis paradigm, in which RBCs are first subjected to a predefined thermal condition and subsequently evaluated in a separate measurement step. Such post-treatment measurements provide only static snapshots and cannot capture the onset, rate, or progression of thermal alterations. In addition, separate sampling introduces variability and prevents tracking of the same RBC population over time. As a result, the dynamic evolution from early functional change to severe thermal injury remains poorly resolved. Thus, there is a need for a platform that can continuously monitor hemorheological changes in the same RBCs under ongoing thermal stress in circulating conditions.

To overcome these limitations, the present study introduces a microfluidic platform that enables continuous and time-resolved monitoring of thermally induced changes in RBC aggregation during closed-loop circulation. The system integrates a peristaltic pump, a thermal mixer, a fluidic stabilizer (FS), and a microfluidic device into a single closed circulation loop. The RBC suspension continuously circulates through the system while being exposed to a controlled temperature, allowing thermal exposure and hemorheological measurement to be performed simultaneously. The thermal mixer provides continuous thermal exposure and suppresses RBC sedimentation, whereas the microfluidic device creates suitable flow conditions for optical quantification of RBC aggregation. Because the same RBC population repeatedly travels through both the heating and sensing regions, temporal changes in the aggregation index (AI) can be tracked without repeated sampling or interruption of circulation. This integrated approach enables direct observation of when aggregation behavior begins to change, how rapidly the change develops, and how it progresses with increasing thermal exposure. Thus, the proposed system provides a dynamic assessment of temperature-dependent and time-dependent RBC alterations rather than a single post-treatment measurement.

Table 1 compares the main features of the present platform with those of previous microfluidic systems. The earlier studies demonstrated hemorheological monitoring during closed-loop circulation [37,38], attenuation of flow fluctuations using air compliance, and RBC aggregation measurement under continuous flow [39,40]. Additional studies evaluated RBCs after thermal pretreatment, or measured AI together with blood viscosity or a viscosity-sensitive time constant during syringe-driven blood delivery [32,33,41].

The proposed platform provides several advantages over conventional thermal-damage assays and existing microfluidic methods. First, it enables continuous, time-resolved tracking of RBC aggregation during ongoing circulation, overcoming the limitation of conventional assays that typically provide only discrete measurements [42]. Second, the same RBC population is repeatedly monitored throughout the experiment, minimizing variability associated with repeated sampling, transfer, and handling. Third, aggregation is quantified without interrupting blood flow, thereby preserving the shear-dependent environment that directly governs RBC–RBC interactions [2,42]. Moreover, thermal exposure, flow stabilization, sedimentation suppression, closed-loop circulation, and microfluidic sensing are integrated within a single platform, allowing hemorheological responses to be evaluated under well-controlled dynamic conditions. Most importantly, this integrated approach enables direct observation of the onset and progression of functional RBC alterations during thermal exposure, providing information that cannot be readily obtained from conventional post-treatment measurements [43,44].

## 2. Materials and Methods

### 2.1. Microfluidic Chip Fabrication and Multiscale Fluidic System

**Figure 1:**
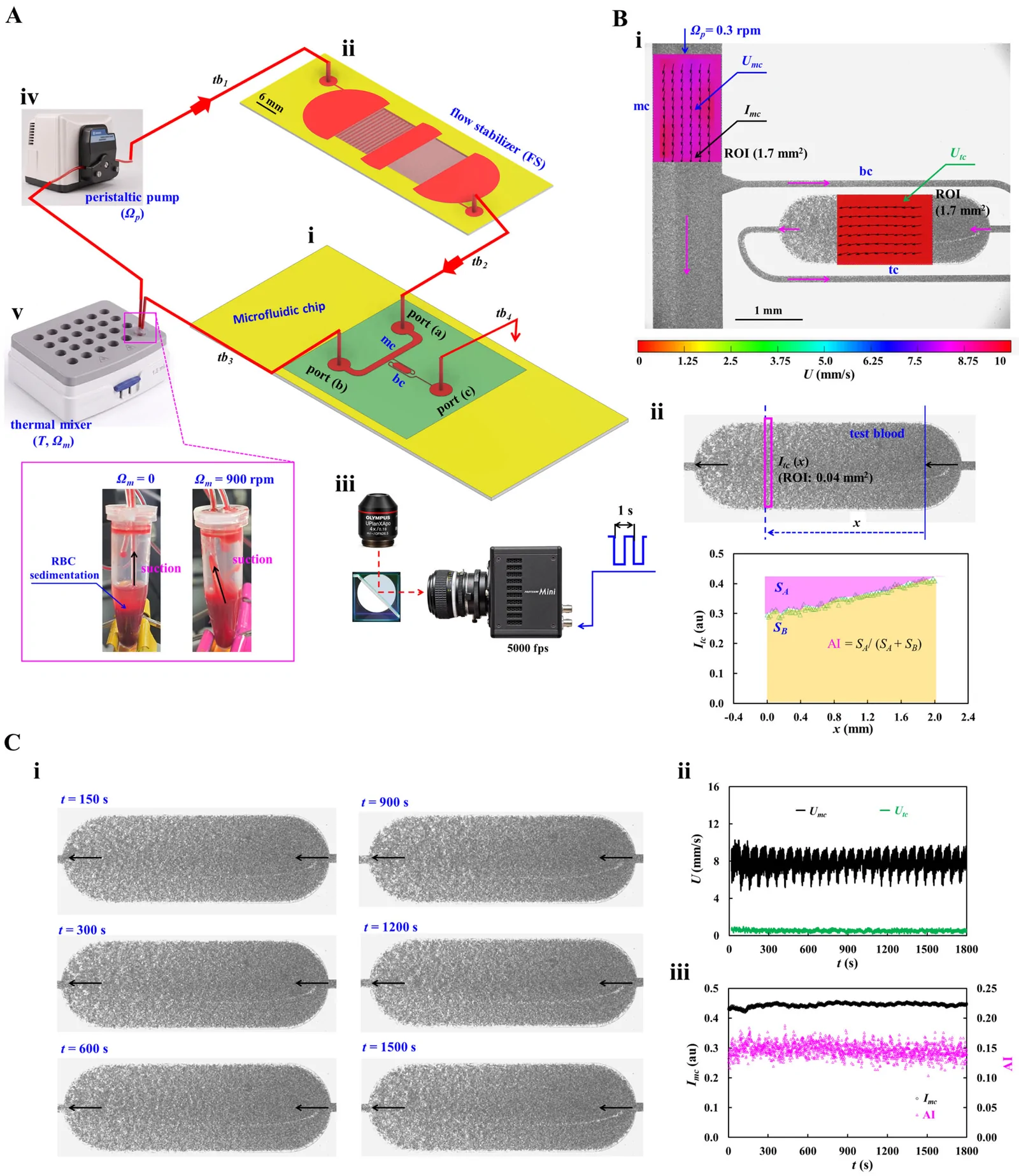
Proposed method for probing thermally induced red blood cell alteration using a multiscale fluidic system. (**A**) Schematic illustration of the experimental setup consisting of a microfluidic device, a fluidic stabilizer (FS), a peristaltic pump, a thermal mixer, and a microscopic imaging system. (**i**) The microfluidic device comprised three ports (a, b, and c), a main channel (mc), and a bifurcation channel (bc). The bc consisted of a front narrow channel, a test chamber (tc), and a rear narrow channel connected in series. (**ii**) The FS comprised an inlet, two semicircular chambers (radius = 10 mm), upstream and downstream pillar channels, a rectangular connector, and an outlet. (**iii**) The microscopic imaging system consisted of a microscope equipped with a 4× objective lens (NA = 0.1), a high-speed camera capable of recording at 5000 frames per second (fps), and an external trigger with a 1 s interval. (**iv**) The peristaltic pump was operated at a constant rotational speed (*Ω_p_*) to circulate the blood sample through the closed-loop system. (**v**) Herein, 1 mL of test blood was transferred into a 1.5 mL microtube, which was then fully inserted into the designated hole of the heating block to ensure stable positioning and efficient heat transfer during thermal treatment. The thermal mixer was then operated at a prescribed vibration speed (*Ω_m_*) to minimize RBC sedimentation within the tube, while a constant temperature (*T*) was maintained to impose the specific thermal shock on RBCs. (**B**) Quantification of blood velocity and image intensity in the main channel and test chamber. (**i**) Blood velocity fields were measured within a selected ROI (1.7 mm^2^) in the main channel (*U_mc_*) and test chamber (*U_tc_*). Purple arrows indicate the flow direction in microfluidic channels. (**ii**) Spatial distribution of (*I_tc_* [*x*]) was measured within a small ROI (0.04 mm^2^) in the test chamber. The distance (x) was defined from the baseline of the downstream semicircle. Black arrows indicate blood flow from right to left. Two area values (*S_A_*, *S_B_*) obtained from the spatially varying *I_tc_* curve were used to calculate the RBC aggregation index as AI = *S_A_*/(*S_A_* + *S_B_*). (**C**) Preliminary evaluation of the proposed method. Test blood (Hct = 30%) was prepared by suspending normal RBCs in dextran solution (4%) and placed in a microtube. The peristaltic pump and mixer were operated at *Ω_p_* = 0.3 rpm and *Ω_m_* = 900 rpm, respectively, while the mixer temperature was maintained at 25 ◦C. (**i**) Representative microscopic images captured at specific times (*t* = 150, 300, 600, 900, 1200, and 1500 s). Black arrows indicate blood flow direction in the test chamber. (**ii**) Time-dependent *U_mc_* and *U_tc_*. (**iii**) Time-dependent changes in *I_mc_* and AI. Herein, the *I_mc_* was obtained from the ROI (1.7 mm^2^) selected in the main channel.

As shown in Figure 1, a multiscale fluidic system was developed for continuous, real-time monitoring of thermally induced RBC damage based on changes in RBC aggregation during closed-loop circulation.

Figure 1A shows a schematic illustration of the multiscale fluidic system consisting of a microfluidic chip, a fluidic stabilizer (FS), a peristaltic pump, a thermal mixer, and a microscopic imaging system. To assess RBC aggregation under continuous blood flow, the previously developed microfluidic chip [33,41] was modified and integrated into a closed-loop circulation system. As shown in Figure 1A(i), the microfluidic device consisted of three ports (a, b, and c), a main channel (mc; width = 1 mm, length = 14.48 mm), and a bifurcation channel (bc). The bc consisted of a front narrow channel (width = 0.1 mm, length = 4.93 mm), a test chamber (tc; width = 1 mm, length = 3 mm), and a rear narrow channel (width = 0.1 mm, length = 8.85 mm) connected in series. To regulate backflow generated in a peristaltic pump, as shown in Figure 1A(ii), the FS consisted of an inlet, two semicircular chambers (radius = 10 mm), upstream and downstream pillar channels (width = 0.05 mm, length = 10 mm, and *N* = 100), a rectangular connector (width = 20 mm, length = 6 mm), and an outlet. Its chambers and pillar channels were incorporated to provide fluidic compliance and hydraulic resistance for attenuating pump-induced flow disturbances. Its performance was evaluated experimentally, but alternative geometries were not systematically compared. The channel depth of the microfluidic chip and FS was fixed at *h* = 0.05 mm.

The microfluidic device was prepared using a PDMS-based soft-lithography process. A silicon wafer containing the channel structures was first fabricated by photolithographic patterning followed by deep reactive ion etching. Sylgard 184 PDMS precursor and curing agent (Dow Corning, Midland, MI, USA) were combined at a mass ratio of 10:1 and thoroughly mixed. The mixture was placed under vacuum for 1 h to remove trapped air bubbles and then poured onto the patterned silicon mold. After thermal curing at 65 °C for 2 h, the cured PDMS layer was carefully separated from the mold and cut to the desired size. Three ports (2 mm diameter) were subsequently created using a biopsy punch. The patterned PDMS layer and glass substrate were treated with oxygen plasma and then immediately bonded together. The bonded device was then heated at 120 °C for 10 min to reinforce the PDMS–glass interface. The fluidic stabilizer (FS) was fabricated following the same procedure. Two 2 mm diameter openings were formed at the inlet and outlet of the FS to allow connection with the circulation tubing.

As illustrated in Figure 1A(iii), the microfluidic device was positioned on the stage of an inverted microscope (IX81, Olympus, Tokyo, Japan) fitted with a 4× objective lens (NA = 0.1). Blood-flow images were recorded with a high-speed camera (FASTCAM MINI, Photron, Tokyo, Japan) at a frame rate of 5000 frames per second (fps). Image recording was initiated periodically at one-second intervals using an external trigger signal.

**Figure A1:**
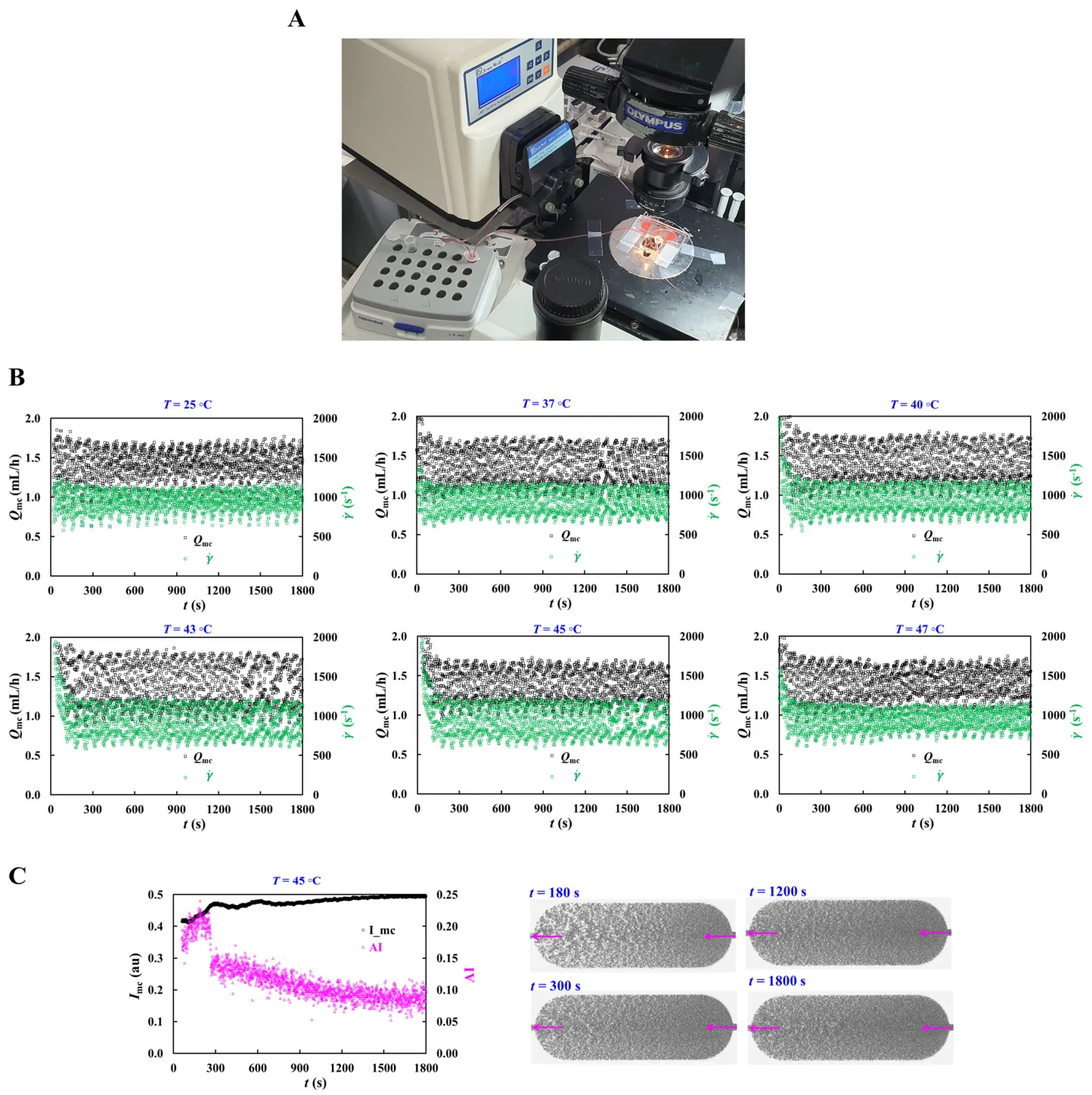
Time-lapse blood flow and blood flow images over heat-exposure temperature. (**A**) Photograph of the experimental setup, showing the peristaltic pump, thermal mixer with a blood-containing microtube, and microfluidic chip and flow stabilizer mounted on the microscope stage for continuous optical monitoring during blood recirculation. (**B**) Time-dependent *Q*_mc_ and $\dot{\gamma }$ with respect to heat-exposure temperature. (**C**) Representative blood-flow images acquired at *t* = 180, 300, 1200, and 1800 s for *T* = 45 °C.

As shown in Figure 1A(iv), the peristaltic pump (JP-100, Jenie Well, Seoul, Republic of Korea) was operated at a constant rotational speed of *Ω_p_* = 0.3 rpm to continuously circulate the blood sample through the closed-loop system. The microfluidic chip, FS, peristaltic pump, and thermal mixer were connected in series using four tubing segments: *tb*_1_ (i.d. = 0.05 mm, *L*_1_ = 600 mm), *tb*_2_ (i.d. = 0.05 mm, *L*_2_ = 50 mm), *tb*_3_ (i.d. = 0.05 mm, *L*_3_ = 300 mm), and *tb*_4_ (i.d. = 0.025 mm, *L*_4_ = 200 mm). As shown in Figure 1A(v), a 1.5 mL microtube was placed in the heating block of a thermal mixer (ThermoMixer C, Eppendorf SE, Hamburg, Germany). As shown in Figure A1A (Appendix A), the microtube was sealed after insertion into the thermal mixer. The thermal mixer was allowed to reach the target temperature (*T*). The pump was then operated to fill the entire circulation loop with blood. The pump was temporarily switched off, and complete cessation of flow in the microfluidic chip was confirmed. The mixing speed (*Ω_m_* = 900 rpm) was subsequently set to a constant value, after which the pump was restarted to initiate blood circulation and AI monitoring, when *t* = 0 was defined. Thermal exposure occurred in the microtube installed in the thermal mixer. After leaving the microtube, blood passed through tubing exposed to the surrounding environment before reaching the microfluidic chip, where AI was measured under ambient conditions of 25 °C. Thus, thermal exposure and optical measurement occurred at separate locations, and the microfluidic chip was not heated to the mixer setpoint. The lower panel showed the effect of the mixer vibration on RBC sedimentation in the microtube. At the end of the experiment, clear RBC sedimentation was observed without vibration (*Ω_m_* = 0), whereas RBC sedimentation was effectively suppressed at *Ω_m_* = 900 rpm.

To reduce nonspecific protein adsorption, all blood-contacting surfaces, including the tubing, microfluidic chip, and FS, were treated with 0.2% bovine serum albumin (BSA) for 10 min [45]. The fluidic pathways were then rinsed with 1× PBS before blood infusion. All experiments were carried out at a room temperature of 25 °C.

### 2.2. Quantification of Blood Velocity, Image Intensity, and RBC Aggregation Index

As illustrated in Figure 1B, local flow conditions and RBC aggregation within the microfluidic channels were evaluated using blood image-based analysis. Blood velocities in the main channel and test chamber were measured to calculate the corresponding local shear rates. In addition, temporal changes in image intensity were analyzed in both regions to monitor variations in blood flow and to determine the RBC aggregation index (AI) in the test chamber.

First, as illustrated in Figure 1B(i), blood flowed downstream through the main channel, while a portion of the blood flow branched into the bifurcation channel at the junction. The purple arrows indicate the direction of blood flow within the microfluidic networks. To determine the local shear-rate conditions in the main channel and test chamber, time-resolved micro-particle image velocimetry (PIV) was employed to obtain velocity fields in both channels [46]. Two regions of interest (ROIs), each with an area of 1.7 mm^2^, were defined: one in the upstream straight section of the main channel, and the other in a straight uniform section of the test chamber. Blood-flow images acquired at one-second intervals were analyzed using PIVlab software (Version 3.14, William Thielicke, Germany) [46]. Velocity fields were calculated using an interrogation window of 143 × 143 µm^2^ with 50% overlap. Abnormal velocity vectors were corrected using local median and standard-deviation filtering. With the present optical imaging system [41], the recorded image intensity reflected the combined contribution of RBCs distributed throughout the channel depth. Thus, the resulting velocity vectors were then spatially averaged over each ROI to obtain the time-dependent mean velocities in the main channel and test chamber, denoted as *U_mc_* and *U_tc_*, respectively.

Second, to consider only RBCs in the blood flow, before intensity analysis, the initial background image was subtracted from each acquired image to reduce stationary background contributions. The resulting background-corrected images were analyzed using MATLAB (version 2026b, MathWorks, Natick, MA, USA). To monitor variations in blood flow, as illustrated in Figure 1B(i), a specific ROI (1.7 mm^2^) was defined in the upstream section of the main channel. The mean image intensity within this ROI was calculated as *I_mc_* by spatially averaging all image intensities.

Third, the conventional aggregation index is calculated from the temporal change in image intensity after abruptly stopping a high-shear flow. In the continuous-flow approach, RBCs were disaggregated in the high-shear main channel and subsequently underwent aggregation in the low-shear test chamber. The resulting spatial intensity profile therefore reflected the progressive aggregation of RBCs as they traveled through the chamber [41]. RBC aggregation in the test chamber was induced by creating a substantial difference in hydraulic resistance between the bifurcation channel and the main channel. The hydraulic resistance of the bifurcation channel was approximately 18.76 times greater than that of the downstream section of the main channel (*R_bc_*/*R_mc_* = 18.76) [41], where *R_bc_* and *R_mc_* represented the hydraulic resistances of the bifurcation channel and the downstream main channel, respectively. At the prescribed inlet flow rate, RBCs remained fully disaggregated while traveling through the main channel because the local shear rate exceeded 1000 s^−1^. Approximately 95% of the incoming flow continued downstream along the main channel, whereas the remaining 5% entered the bifurcation channel. Within the narrow sections of the bifurcation channel, the shear rate remained above 100 s^−1^, thereby maintaining the RBCs in a disaggregated state. In contrast, the substantially reduced shear rate in the wider test chamber (<100 s^−1^) provided a low shear-rate environment, which promoted RBC aggregation, whereas RBC aggregates were progressively dispersed with increasing shear forces [2,47,48,49]. As shown in the upper panel of Figure 1B(ii), blood flowed from left to right through the test chamber, as indicated by the black arrow. RBC aggregation gradually increased along the chamber, causing the image intensity to decrease toward the downstream region. When the blood entered the rear narrow channel, the shear rate increased above 1000 s^−1^, causing the RBC aggregates to break apart. A small ROI (0.04 mm^2^) was selected within the straight portion of the test chamber and moved stepwise from right to left along the *x*-axis. At each location, the pixel intensities within the ROI were spatially averaged to obtain *I_tc_* (*x*). The measured *I_tc_* (*x*) progressively decreased over a 2 mm distance, reflecting the gradual development of RBC aggregation within the test chamber. Following the conventional definition of the RBC aggregation index (AI) [50,51,52], two characteristic areas (*S_A_*, *S_B_*) were determined from the spatial variation in *I_tc_*. The RBC aggregation index was subsequently calculated as AI = *S_A_*/(*S*_A_ + *S*_B_).

**Figure A2:**
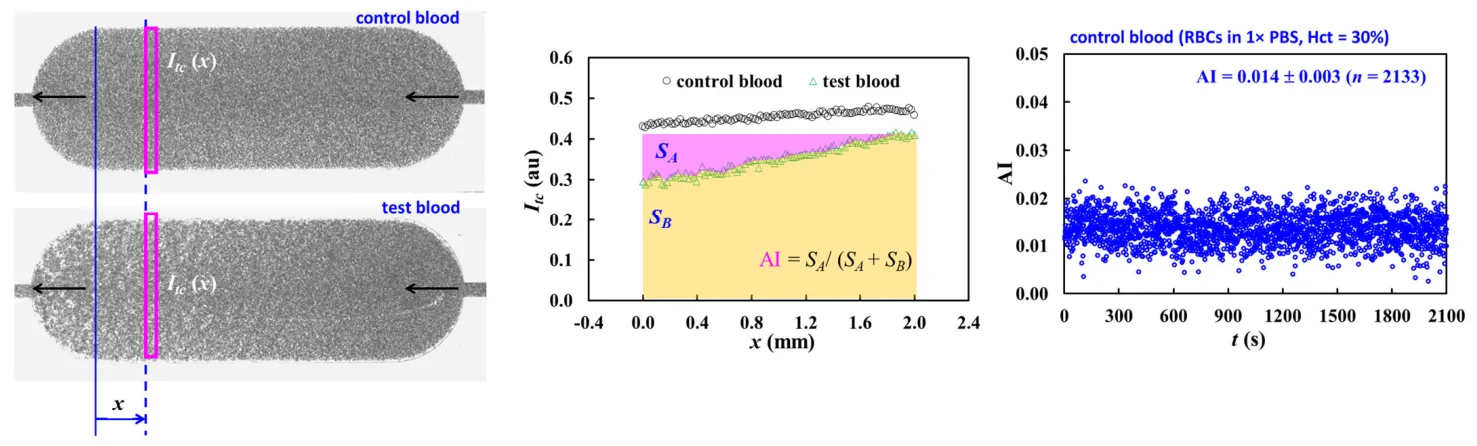
Comparison of control and test blood and characterization of the baseline aggregation index (AI). Left panel: Representative images of control and test blood in the test chamber. Middle panel: Spatial intensity profiles for calculating *S*_A_ and *S*_B_. Right panel: Temporal variation in AI for the control suspension (RBCs in 1× PBS, Hct = 30%), yielding a mean ± standard deviation of 0.014 ± 0.003 from *n* = 2133 temporal measurements.

As a preliminary demonstration, blood conditions that favor RBC sedimentation were intentionally selected to evaluate whether the proposed closed-loop system could suppress sedimentation while maintaining consistent AI measurements. A relatively low hematocrit was used because RBC sedimentation generally increases as hematocrit decreases, whereas a high dextran concentration was selected to enhance RBC aggregation and thereby promote sedimentation [53,54,55,56]. Test blood with a hematocrit of 30% was prepared by suspending normal RBCs in a 4% dextran solution and then loaded into a microtube. During the experiment, the peristaltic pump and thermal mixer were operated at *Ω_p_* = 0.3 rpm and *Ω_m_* = 900 rpm, respectively, while the mixer temperature was kept constant at 25 °C. As shown in Figure 1C(i), microscopic images were acquired at six representative time points (*t* = 150, 300, 600, 900, 1200, and 1500 s). The black arrows indicate the direction of blood flow through the test chamber. From these images, the time-dependent mean velocities in the main channel (*U_mc_*) and test chamber (*U_tc_*) were determined over the 1800 s circulation period. As presented in Figure 1C(ii), *U_mc_* remained relatively stable, with only limited backflow owing to the damping effect of the fluidic stabilizer (FS). The flow rates in the main channel upstream of the bifurcation (*Q*_mc_) and in the test channel (*Q*_tc_) were estimated to be 1.41 ± 0.18 and 0.08 ± 0.03 mL/h, respectively. Blood returned to the microtube through port (b) at a mean flow rate of *Q*_mc_ − *Q*_tc_ = 1.33 mL/h, while the test-channel outflow was discharged through port (c). Thus, the system recirculated blood with continuous side-stream discharge. The combined nominal internal volume of tb_1_, the flow stabilizer, and tb_2_ was approximately 27.99 µL, corresponding to an estimated transit time of 71.4 s to the chip inlet. Including the main channel and return tubing tb_3_, the nominal transit time from the microtube outlet back to the microtube through port (b) was approximately 75 s. Figure 1C(iii) presents the corresponding temporal variations in *I_mc_* and AI. The translational motion of the thermal mixer effectively prevented RBC sedimentation during circulation, enabling the AI to remain nearly constant over the 1800 s measurement period in the absence of thermal exposure. As shown in Figure A2 (Appendix A), the control suspension (RBCs in 1× PBS, Hct = 30%) exhibited a low baseline AI of 0.014 ± 0.003 (*n* = 2133 temporal measurements), indicating minimal aggregation under the tested conditions. These preliminary results demonstrated that the proposed platform could provide stable and continuous monitoring of blood circulated in a closed-loop fluidic system.

### 2.3. Preparation of Test Blood Sample

This study was performed in accordance with the Declaration of Helsinki and was approved by the Ethics Committee of Chosun University (approval no. 2-1041055-AB-N-01-2026-23). Packed RBCs were supplied by the Gwangju–Chonnam Blood Bank (Gwangju, Republic of Korea) and stored under refrigerated conditions (4 °C) until use. Before each experiment, the RBCs were washed with 1× PBS according to the established preparation protocol [57]. After centrifugation, the buffy coat and supernatant were removed, and the remaining washed RBCs were collected for preparation of the test blood samples.

First, to examine the influence of hematocrit on AI measurements, test blood samples with hematocrit levels of 30% and 50% were prepared by suspending normal RBCs in a 4% dextran solution. The hematocrit of the test blood was set volumetrically, based on the RBC volume fraction. The dextran solution was prepared by dissolving dextran powder (Leuconostoc spp., molecular weight 450–650 kDa; Sigma-Aldrich, St. Louis, MO, USA) in 1× PBS. Second, to investigate the effect of the suspending medium on AI measurements, test blood with a hematocrit of 30% was prepared using dextran solutions with concentrations ranging from 1% to 4% (*C*_dex_ = 1~4%). Finally, to examine the effect of temperature on AI measurements, test blood (Hct = 30%) was prepared in 4% dextran solution and exposed to 25, 37, 40, 43, 45, and 47 °C using the thermal mixer. For each temperature condition (25~47 °C), test blood (1 mL) was prepared at room temperature and loaded into a microtube. The thermal mixer was set to the target temperature before the experiment began. Each test blood sample was measured at only one temperature, without sequential exposure to other temperatures. After each experiment, the microfluidic chip, tubing, and flow stabilizer were rinsed with 1× PBS to remove remaining RBC suspension and reduce carryover to the next experiment. At least two experiments were conducted at each temperature to assess reproducibility.

## 3. Results and Discussion

### 3.1. Quantitative Evaluation of Flow Regulation Using a Flow Stabilizer

**Figure 2:**
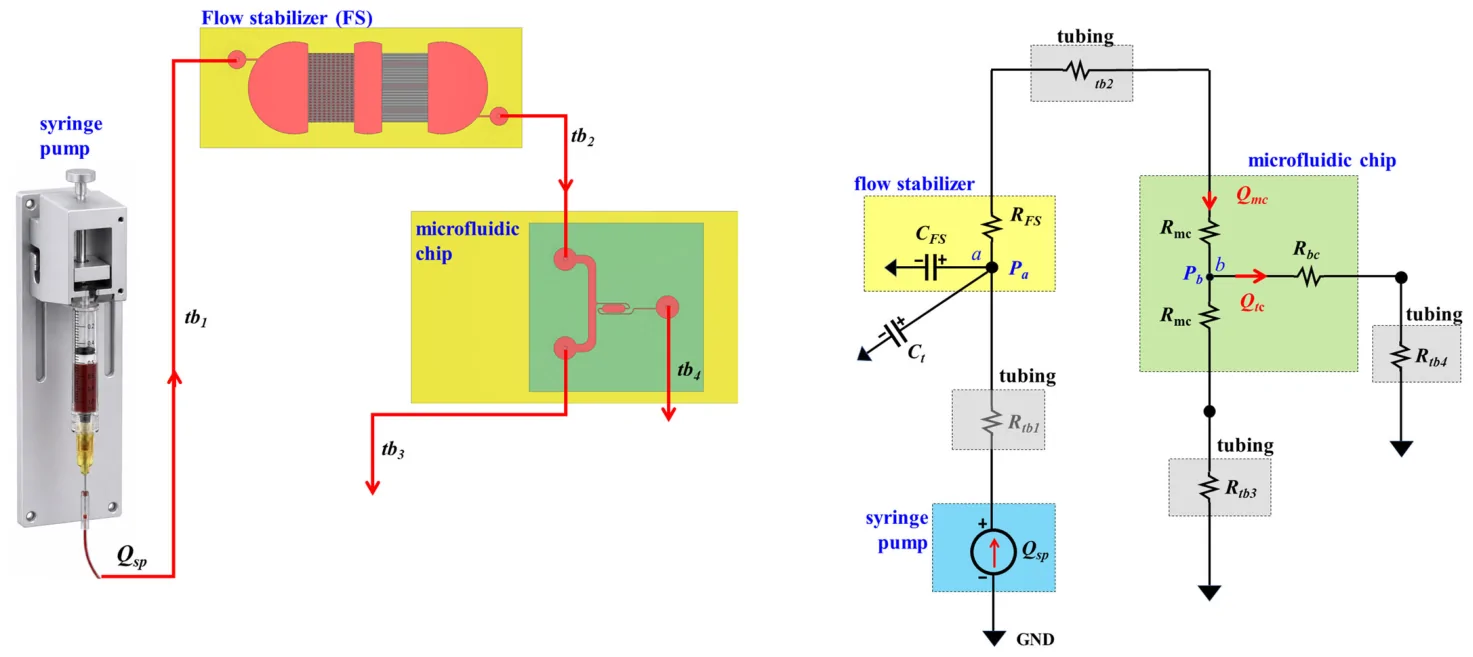
Fluidic circuit model for evaluating the effect of the FS on flow regulation. The left panel shows a schematic of the experimental setup, in which the flow rate is prescribed as *Q_sp_* = *Q*_0_ + *Q*_1_ sin (2π*t*/*T_p_*). The right panel shows the corresponding circuit model of the experimental setup. The fluidic circuit was composed of fluidic resistances (tubing: *R*_*tb*1_~*R*_*tb*4_, *Fs*: *R_FS_*, and channel: *R_mc_*, *R_bc_*), compliances (FS: *C_FS_*, tubing: *C_t_*), and flow rate (*Q_sp_*). The ‘▼’ denotes zero pressure as boundary condition.

In this study, a flow stabilizer (FS) was introduced to attenuate pulsatile flow fluctuations generated by the pulsatile pump and thereby provide a more stable flow condition in the microfluidic system. As shown in Figure 2, to quantitatively characterize the flow-regulating performance of the FS, the pulsatile pump was replaced with a syringe pump (neMESYS, CETONI GmbH, Korbussen, Germany), which enabled precise control of flow rates. By generating prescribed steady and sinusoidally varying flow rates, the dynamic response of the fluidic system could be systematically examined with and without the FS. The effect of the FS was quantitatively evaluated based on the transient flow response, characterized by the time constant [58], and the attenuation of pulsatile flow fluctuations [59,60,61,62,63,64]. In addition, the flow rates and corresponding shear rates in the microfluidic channels were estimated from the velocity measurements obtained by the micro-PIV technique [65].

As shown in the left panel of Figure 2, the experimental setup consisted of a syringe pump, a fluidic stabilizer (FS), and a microfluidic chip, which were interconnected using flexible tubing. To visualize the fluid motion for micro-PIV analysis [66], a tracer suspension was prepared by adding normal RBCs (30 μL) to 30% glycerin solution (1000 μL) [32,67]. The added RBCs served as tracer particles for determining the velocity fields in the microfluidic channels. The test fluid was supplied using a syringe pump to precisely control the imposed flow rate. To determine the time constant (*λ*) from transient flow response, the flow rate was first maintained at a constant value (*Q*_0_) and then abruptly reduced to zero. Additionally, to assess the attenuation of periodic flow fluctuations by the FS, a sinusoidally varying flow rate was imposed as *Q_sp_* = *Q*_0_ + *Q*_1_sin(2π*t*/*T_p_*), where *Q*_0_, *Q*_1_, and *T_p_* represent the mean flow rate, oscillation amplitude, and pulsation period, respectively. As shown in the right panel of Figure 2**,** an equivalent fluidic circuit was developed to calculate the flow rates and shear rates in the microfluidic channels. The circuit included hydraulic resistances corresponding to the tubing (*R*_*tb*1_~*R*_*tb*4_), flow stabilizer (*R_FS_*), main channel (*R_mc_*), and bifurcation channel (*R_bc_*), together with the compliances of the FS (*C_FS_*) and flexible tubing (*C_t_*). The syringe-pump flow rate is represented by *Q_sp_*, while the symbol ‘▼’ indicates zero pressure. *P_a_* and *P_b_* represent the pressures at the FS and at the bifurcation junction, respectively. *Q_mc_* and *Q_tc_* denote the flow rates through the upstream main channel and the bifurcation channel, respectively. Applying the mass conservation law at two nodes (a, b) [68,69,70], the governing equations can be written as

(1) $$Q_{sp}=(C_{t}+C_{FS})\frac{d}{dt}(P_{a})+\frac{P_{a}-{\;P}_{b}}{R_{FS}+R_{tb2}+{\;R}_{mc}},$$

(2) $$\frac{P_{a}-{\;P}_{b}}{R_{FS}+R_{tb2}+R_{mc}}=\frac{P_{b}}{R_{tb3}+R_{mc}}+\frac{P_{b}}{R_{tb4}+R_{bc}}.$$

Rearranging Equation (2) gives the relationship between *P_a_* and *P_b_* as $P_{a}=\kappa P_{b}$, where κ is defined as

(3) $$\kappa =1+(R_{FS}+R_{tb2}+R_{mc})\times (\frac{1}{R_{mc}+R_{tb3}}+\frac{1}{R_{bc}+R_{tb4}})$$

Defining the left-hand side of Equation (2) as *Q_mc_*, the flow rate in the main channel can be written as,

(4) $$Q_{mc}=\frac{P_{a}-P_{b}}{R_{FS\;}+R_{tb2}+{\;R}_{mc}}.$$

Using Equations (3) and (4), the pressure *P_a_* can be expressed as

(5) $$P_{a}=\frac{\kappa }{\kappa -1}(R_{FS}+R_{tb2}+R_{mc})Q_{mc}.$$

Substituting Equations (4) and (5) into Equation (1), flow rate in the main channel (*Q_mc_*) can be described by the following first-order linear differential equation.

(6) $$\lambda \frac{d}{dt}(Q_{mc})+Q_{mc}=Q_{sp.}$$

The corresponding time constant (λ) was given by

(7) $$\lambda =\frac{\kappa }{\kappa -1}(R_{FS}+R_{tb2}+R_{mc})(C_{t}+C_{FS}).$$

According to Equation (7), the time constant (*λ*) determined the dynamic response of *Q_mc_* and was governed by the hydraulic resistances and compliance elements in the fluidic network.

**Figure 3:**
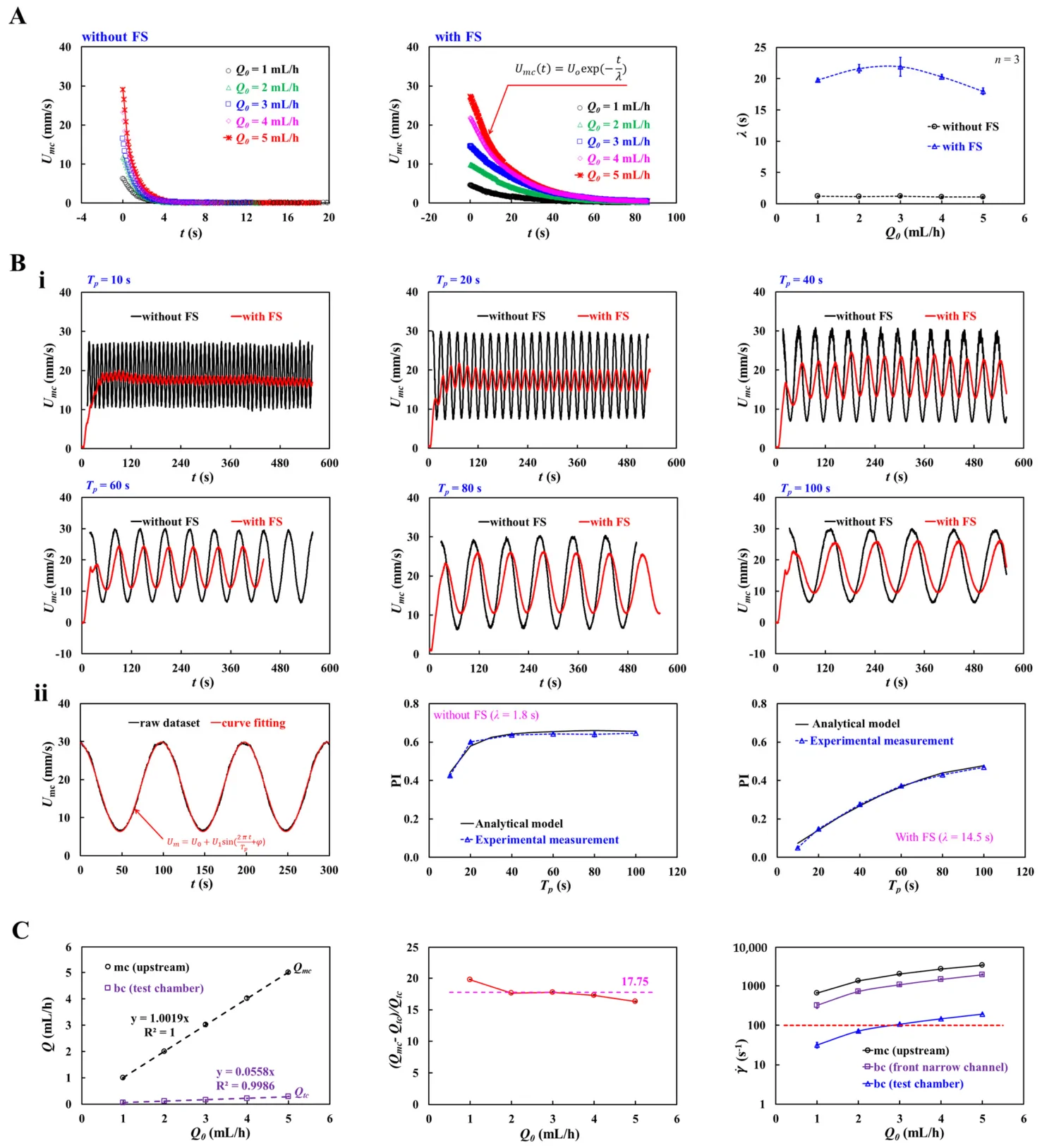
Effect of the flow stabilizer (FS) on flow regulation and shear rate estimation under controlled flow conditions. To visualize fluid velocity fields in microfluidic channels, normal RBCs (30 μL) were added into 30% glycerin solution (1000 μL). Flow rate was controlled precisely using a syringe pump. (**A**) Effect of the plateau flow rate (*Q*_0_) on the time constant (λ). The *Q*_0_ was varied from 1 mL/h to 5 mL/h. The left and middle panels show the transient main channel velocity (*U_mc_*) after the flow rate was changed from *Q*_0_ to zero, without and with the FS. To determine *λ*, the transient response of *U_mc_* was best fitted using a first-order exponential decay model. The right panel compares λ as a function of *Q*_0_, with and without the FS. (**B**) Effect of pulsation period (*T*p) on flow regulation with and without the flow stabilizer (FS). (**i**) Temporal profiles of the main-channel mean velocity (*U*_mc_) at *T*_p_ = 10, 20, 40, 60, 80, and 100 s. (**ii**) Left panel: representative sinusoidal fitting of *U*_mc_ at *T*_p_ = 100 s without the FS. Middle and right panels: comparison of experimentally measured and analytically predicted pulsation indices (PI) as a function of *T*_p_ without the FS (*λ* = 1.8 s) and with the FS (*λ* = 14.5 s). PI was calculated as (*U*_max_ − *U*_min_)/(2*U*_ave_) using the fitted velocity profiles. Experimental values represent the mean ± standard deviation across analyzed pulsation cycles. (**C**) Calculation of shear rates in the microfluidic channels as a function of *Q*_0_. The left panel shows *Q_mc_* and *Q_tc_* with respect to *Q*_0_. The middle panel shows the variation of (*Q_mc_* − *Q_tc_*)/*Q_tc_* with respect to *Q*_0_. The right panel shows the corresponding shear rates in the main channel and bifurcation channel as a function of *Q*_0_.

To evaluate the effects of the plateau flow rate (*Q*_0_) and the fluidic stabilizer (FS) on the transient flow response, the time-dependent *U_mc_* was measured at *Q*_0_ = 1~5 mL/h. As shown in Figure 3A, the left and middle panels present the temporal variations in *U_mc_* after the infusion flow rate was abruptly reduced from *Q*_0_ to zero, without and with the FS, respectively. Incorporation of the FS produced a markedly slower decay in Umc, indicating a substantial increase in the characteristic response time of the fluidic system. The transient decay in *U_mc_* was therefore fitted using a first-order exponential model to determine the time constant (*λ*). The right panel summarizes λ as a function of *Q*_0_ under both conditions. Each measurement was repeated three times (*n* = 3). Without the FS, λ remained low and nearly constant, ranging from 1.09 s to 1.21 s over *Q*_0_ = 1~5 mL/h. In contrast, with the FS, λ increased substantially and ranged from 17.98 s to 21.90 s over *Q*_0_ = 1~5 mL/h. Without the FS, λ changed slightly with respect to *Q*_0_. With the FS, λ was varied with respect to *Q*_0_, showing the highest value at *Q*_0_ = 3 mL/h and the lowest value at *Q*_0_ = 5 mL/h. Overall, the FS increased the time constant by about 17.6 times, indicating that it strongly affected the transient flow response. This result was consistent with the fluidic resistance–compliance (RC) model, in which the characteristic time constant was determined by the hydraulic resistance and compliance of the fluidic system (λ∼RC). Therefore, an increase in fluidic compliance could substantially prolong the transient response [41,71,72,73].

To evaluate the effect of the FS on pulsatile-flow attenuation, the time-dependent *U_mc_* was measured at different pulsation periods. The syringe-pump flow rate was prescribed as *Q_sp_* = 3 + 2 sin ($\frac{2\pi t}{T_{p}}$) mL/h, while the pulsation period (*T_p_*) was varied from 10 s to 100 s. As shown in Figure 3B(i), the FS reduced the amplitude of the pulsatile flow, with a greater reduction observed at shorter *T_p_*. Table A1 (Appendix A) summarizes the mean, standard deviation, and coefficient of variation (COV) of main-channel blood velocity at different pulsation periods. Without the FS, the COV ranged from 30.26% to 45.51%. With the FS, the COV was 4.30%, 10.65%, 19.67%, 26.44%, 30.32%, and 32.94% at pulsation periods of 10, 20, 40, 60, 80 s, and 100 s, respectively. These values corresponded to relative COV reductions of 85.77%, 74.96%, 56.78%, 41.90%, 33.37%, and 26.5%, demonstrating greater attenuation of flow fluctuations at shorter pulsation periods. Mean blood velocities were similar with and without the FS. This behavior was consistent with the low-pass characteristics of a fluidic resistance–compliance circuit, in which higher-frequency flow fluctuations were more strongly attenuated [74,75]. For comparison with the experimental results, Eqn (6) was solved under periodic steady-state conditions, yielding the analytical expression for *Q_mc_* given as

(8) $$Q_{mc}=3+\frac{2}{\sqrt{1+{\left(\frac{2\pi \lambda }{T_{p}}\right)}^{2}}}sin(\frac{2\pi t}{T_{p}}-{tan}^{-1}[\frac{2\pi \lambda }{T_{p}}])\mathrm{mL}/h.$$

According to Equation (8), the amplitude of *Q_mc_* depended on *T_p_* and increased with increasing *T_p_*. This trend agreed with the frequency response of the first-order fluidic low-pass filter, where the output amplitude decreased as the input frequency increased [76,77].

To compare the experimental results with the analytical model, the pulsation index [74] was calculated as PI = 0.5 (*U*_max_ − *U*_min_)/*U*_ave_, where Umax, Umin, and Uave denote maximum, minimum, and mean fluid velocity. The left panel of Figure 3B(i) illustrated the sinusoidal fitting of the main-channel velocity measured at *T*_p_ =100 s without the FS. With the period fixed at 100 s, the velocity data were fitted using *U*_mc_ = *U*_0_ + *U*_1_ sin [2π*t*/*T*_p_ + *ϕ*], where *U*_0_, *U*_1_, and *ϕ* denote the mean velocity, oscillation amplitude, and phase, respectively. The time-dependent Umc was best fitted as *U*_mc_ = 18.11 + 11.7 sin (2π*t*/100 + 1.73) mm/s. The fitted mean velocity and oscillation amplitude were 18.109 and 11.725 mm/s, respectively. The corresponding pulsation index was then calculated as PI = *U*_1_/*U*_0_, yielding approximately 0.65. As shown in the middle panel of Figure 3B, without the FS, PI increased from approximately 0.43 at *T*_p_ =10 s to 0.60 at 20 s and remained near 0.64~0.65 at longer periods. As shown in the right panel of Figure 3B, with the FS, PI increased gradually from approximately 0.05 at *T*_p_ = 10 s to 0.47 at *T*_p_ = 100 s. Thus, the FS reduced pulsatility across all tested periods, with greater attenuation at shorter periods. The analytical model closely reproduced the measured PI trends when effective time constants of 14.5 s with the FS and 1.8 s without the FS were used. These values differed from the corresponding time constants obtained from transient responses, which were 20.31 s and 1.19 s, respectively. The discrepancy suggests that a single time constant does not fully capture both transient relaxation and periodic attenuation in the experimental system. Thus, the effective time constants provide an approximate description of the period-dependent attenuation under the tested conditions. Because they were selected to match the measured PI values, the resulting agreement represents model calibration rather than independent validation based on the transiently measured time constants.

Because the FS sufficiently attenuated the pulsatile flow, the test fluid was subsequently supplied at a constant flow rate (*Q*_0_). Since RBC aggregation was strongly dependent on shear rate, the flow rates and corresponding shear rates in the microfluidic channels were evaluated as a function of *Q*_0_. As shown in the left panel of Figure 3C, both *Q_mc_* and *Q_tc_* increased linearly with respect to *Q*_0_. Linear regression yielded *Q_mc_* = 1.0019 *Q*_0_ (R^2^ = 1.000) and *Q_tc_* = 0.0558 *Q*_0_ (R^2^ = 0.9986), confirming a nearly linear relationship between the imposed flow rate and the flow rates in each channel. The pressure *P_b_* at junction b, where the downstream of main channel connected to the bifurcation channel, can be expressed as

(9) $$P_{b}=(R_{mc}+R_{tb3})(Q_{mc}-Q_{tc})=(R_{bc}+R_{tb4})Q_{tc}.$$

From this relationship, the hydraulic resistance ratio between the downstream of main channel and the bifurcation channel was derived as

(10) $$\frac{R_{bc}+R_{tb4}}{R_{mc}+R_{tb3}}=\frac{Q_{mc}-{\;Q}_{tc}}{Q_{tc}}.$$

To experimentally evaluate this resistance ratio, (*Q_mc_* − *Q_tc_*)/*Q_tc_* was plotted against *Q*_0_, as shown in the middle panel of Figure 3C. This ratio decreased slightly with increasing *Q*_0_, with an average value of 17.75. The corresponding resistance ratio calculated from the analytical model was estimated as *R_bc_*/*R_mc_* = 18.76. The difference between the experimental and analytical values was approximately 5.7%, suggesting a relatively small contribution from the tubing connections and other additional flow resistances. Based on time-dependent *Q_mc_* and *Q_tc_*, shear rates in the main channel and bifurcation were estimated using the analytical expression $\dot{\gamma }=\frac{6\;Q}{w\;h^{2}}$. The right panel of Figure 3C shows the resulting shear rates as a function of *Q*_0_, with the red dashed line indicating the threshold shear rate of 100 s^−1^. The shear rate remained above 100 s^−1^ in the main channel and the narrow sections of the bifurcation channel. In contrast, the shear rate in the test chamber remained below 100 s^−1^ at *Q*_0_ < 3 mL/h. These results indicated that RBC aggregates were expected to be dispersed in the main channel and narrow bifurcation sections, whereas low-shear conditions favorable for RBC aggregation were selectively established in the test chamber at *Q*_0_ < 3 mL/h.

From the experimental investigation, the FS effectively stabilized the flow by attenuating pulsatile fluctuations and increasing the transient response time of the fluidic circuit. Under steady-flow conditions, the regulated flow produced predictable flow-rate distributions and established a low-shear region in the test chamber, providing suitable conditions for reliable RBC aggregation measurement.

### 3.2. Validation of the Flow Stabilizer for Suppressing Backflow and Maintaining Stable Flow

After the performance of the flow stabilizer (FS) was characterized using a syringe pump, its effectiveness was further evaluated under a closed-loop circulation condition driven by a peristaltic pump, as illustrated in Figure 1A. Because peristaltic pumping inherently generated periodic flow fluctuations and transient backflow [60,62,78,79], the FS was tested at different pump speeds (*Ω_p_*) to assess backflow suppression and flow stability. Test fluids (i.e., 30% glycerin solution, control blood: RBCs suspended in 1× PBS [Hct = 50%]) were selected in this study. With regard to the control blood, RBCs were suspended in 1× PBS to minimize RBC aggregation and thereby evaluate the hydrodynamic behavior of circulating blood independently of aggregation [80].

**Figure 4:**
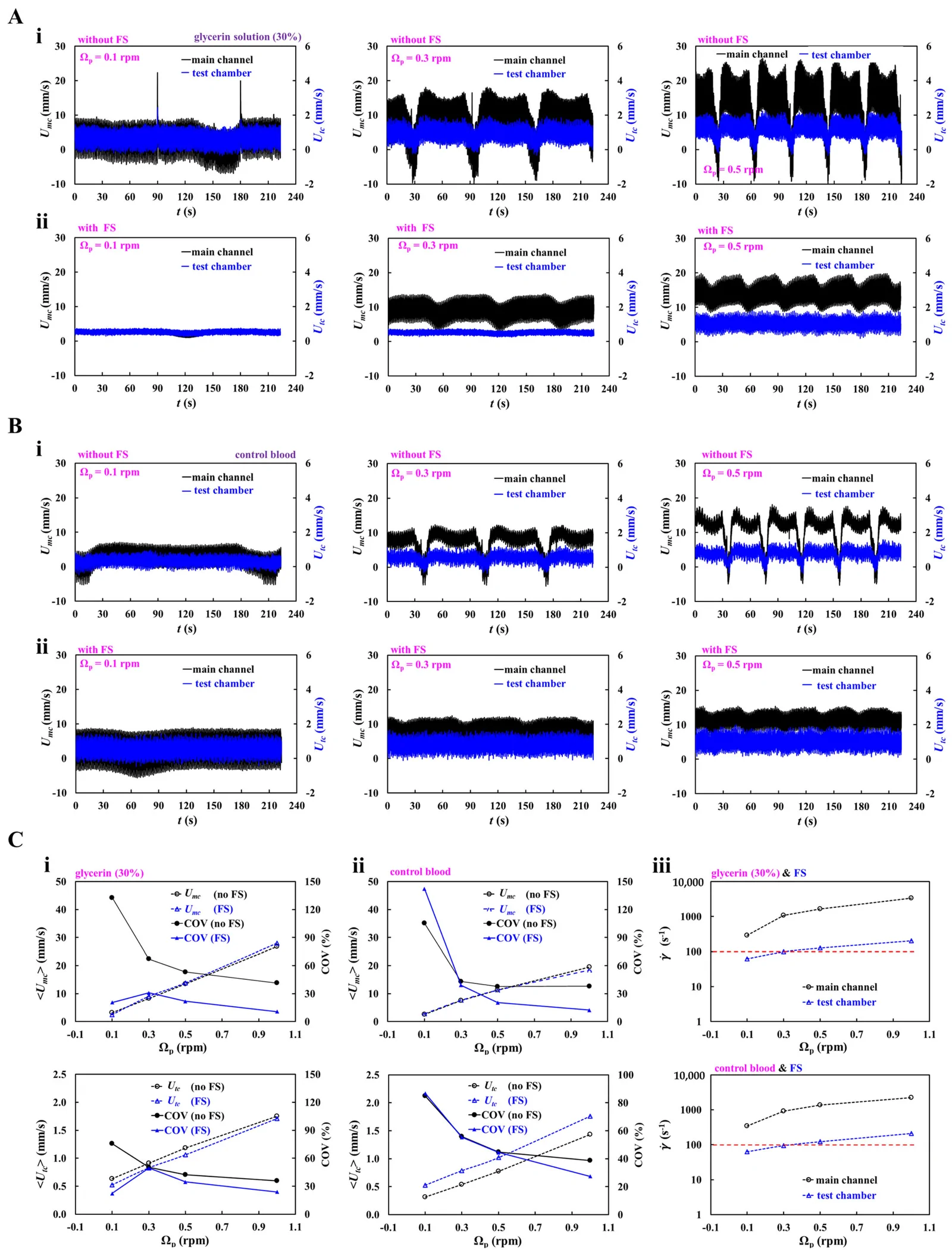
Validation of the flow stabilizer (FS) for suppressing backflow and maintaining stable flow in microfluidic channels. (**A**) Effect of the FS on flow stabilization using glycerin solution (30%) as the test fluid. (**i**) Time-dependent *U_mc_* and *U_tc_* with respect to *Ω_p_* = 0.1~0.5 rpm, without the FS. (**ii**) Time-lapse *U_mc_* and *U_tc_* with respect to *Ω_p_* = 0.1~0.5 rpm, with the FS. (**B**) Effect of the FS on flow stabilization using control blood (normal RBCs suspended in 1× PBS, Hct = 50%). (**i**) Time-dependent *U_mc_* and *U_tc_* with respect to *Ω_p_* = 0.1~0.5 rpm, without the FS. (**ii**) Time-lapse *U_mc_* and *U_tc_* with respect to *Ω_p_* = 0.1~0.5 rpm, with the FS. (**C**) Effect of the FS on flow stability and shear rate estimation in the main channel and test chamber. (**i**) Mean velocities (<*U_mc_*>, <*U_tc_*>) and coefficients of variation (COVs) as a function of pump speed (*Ω_p_* = 0.1~1.0 rpm) for 30% glycerin solution. (**ii**) Variations of <*U_mc_*>, <*U_tc_*>, and their COVs as a function of pump speed for control blood. (**iii**) Estimation of shear rate ($\dot{\gamma }$) in the main channel and test chamber with respect to *Ω_p_* for both test fluids. The purple line indicates the upper shear rate threshold ($\dot{\gamma }$ = 100 s^−1^) for promoting RBC aggregation in the test chamber.

As shown in Figure 4A(i), the time-dependent velocities in the main channel (*U_mc_*) and test chamber (*U_tc_*) were measured for a 30% glycerin solution at *Ω_p_* = 0.1, 0.3, and 0.5 rpm without the FS. Both *U_mc_* and *U_tc_* exhibited clear periodic fluctuations accompanied by repeated backflow, and the oscillation period became shorter as *Ω_p_* increased. In contrast, when the FS was incorporated into the fluidic circuit as shown in Figure 4A(ii), backflow was effectively suppressed over the tested pump-speed range. The magnitude of the pulsatile velocity fluctuations was also markedly reduced. These results demonstrated that the FS effectively suppressed pump-induced backflow and attenuated pulsatile flow, thereby providing more stable fluid circulation.

After replacing the 30% glycerin solution with control blood, the effects of the FS on blood-flow stability were further evaluated. As shown in Figure 4B(i), without the FS, both *U*_mc_ and *U_tc_* exhibited pronounced periodic fluctuations and repeated backflow at *Ω_p_* = 0.1, 0.3, and 0.5 rpm. These pulsatile blood-flow patterns were similar to those observed with the 30% glycerin solution. Such periodic fluctuations and backflow were characteristic of fluid delivery driven by peristaltic pumps, which had been reported in microfluidic blood-circulation systems [60,78,81]. In contrast, as shown in Figure 4B(ii), incorporation of the FS markedly suppressed backflow and reduced the magnitude of velocity fluctuations over the entire range of pump speeds. These results confirm that the FS could stabilize peristaltic-pump-driven flow for glycerin solution and blood effectively [60]. These results confirm that the FS can effectively stabilize peristaltic-pump-driven flow for both Newtonian test fluids and RBC suspensions.

To quantitatively evaluate the effectiveness of the FS in attenuating pulsatile flow, the mean velocities and coefficients of variation (COVs) were calculated from the time-dependent *U*mc and *U*tc data. For the 30% glycerin solution, Figure 4C(i) shows the mean velocities (<*U*_mc_> and <*U*_tc_>) and corresponding COVs at *Ω_p_* = 0.1, 0.3, 0.5, and 1.0 rpm. Both mean velocities increased approximately linearly with respect to *Ω*p, with similar values obtained with and without the FS. In contrast, incorporation of the FS markedly reduced the COVs of both *U*_mc_ and *U_tc_*. These results demonstrated that the FS effectively attenuated pulsatile flow fluctuations without substantially altering the mean flow velocity. For control blood, Figure 4C(ii) summarizes the mean velocities (<*U_mc_*> and <*U_tc_*>) and their corresponding COVs as a function of pump speed. The mean velocity of *U_mc_* (<*U_mc_*>) remained nearly unchanged by the installation of the FS, whereas <*U_tc_*> increased in the presence of the FS. More importantly, the FS substantially reduced the COV of *U*_mc_ at *Ω_p_* = 0.5 and 1.0 rpm, indicating effective attenuation of pulsatile blood flow at higher pump speeds. A reduction in the COV of *U_tc_* was also observed at *Ω_p_* = 1.0 rpm. In contrast, at the lowest pump speed (*Ω_p_* = 0.1 rpm), the FS increased the flow fluctuations of *U_mc_*. The reduced stabilization observed at *Ω_p_* = 0.1 rpm may be attributed to the relatively long pulsation period of the peristaltic pump. Because a compliance-based flow stabilizer behaved as a low-pass fluidic filter, slowly varying flow disturbances were less effectively attenuated than higher-frequency pulsations [60,78,82]. As the pump speed increased, the pulsation period became shorter, resulting in more effective attenuation by the FS. Overall, the FS provided effective stabilization of circulating blood at *Ω_p_* ≥ 0.3 rpm, with its attenuation performance becoming more pronounced at higher pump speeds.

With the FS incorporated into the closed-loop fluidic circuit, the shear-rate conditions required for RBC aggregation were evaluated as a function of pump speed (*Ω_p_*). As shown in Figure 4C(iii), the shear rates in the main channel and test chamber were plotted against *Ω_p_* for 30% glycerin solution and control blood. The horizontal line at $\dot{\gamma }$ = 100 s^−1^ indicates the approximate upper shear-rate limit for RBC aggregation [83,84,85,86]. RBC aggregation was favored at low shear rates, whereas aggregates were gradually dispersed as the shear rate approached or exceeded 100 s^−1^. In the main channel, the shear rate remained sufficiently high over the tested pump-speed range, thereby maintaining RBCs in a disaggregated state. In contrast, the shear rate in the test chamber decreased below 100 s^−1^ at *Ω_p_* ≤ 0.3 rpm, providing favorable hydrodynamic conditions for RBC aggregation. These results identified *Ω_p_* ≤ 0.3 rpm as the appropriate operating range for inducing RBC aggregation selectively in the test chamber while maintaining RBC disaggregation in the main channel.

From the experimental investigation, the FS suppressed backflow effectively, and it reduced pulsatile flow fluctuations for 30% glycerin solution and control blood, while maintaining the mean flow rate. The stabilizing effect became evident at *Ω_p_* ≥ 0.3 rpm, demonstrating that the FS could provide stable circulation under peristaltic pumping. At *Ω_p_* = 0.3 rpm, the shear rate was high enough to prevent RBC aggregation in the main channel but low enough to promote RBC aggregation in the test chamber.

### 3.3. Effect of RBC Sedimentation on AI and Its Suppression for Stable AI Measurement

RBC sedimentation during blood circulation could cause a gradual change in the hematocrit of blood entering the microfluidic chip [2,32,87]. This variation may alter the measured aggregation index (AI) and reduce the reliability of continuous AI monitoring [88,89]. In this section, RBC sedimentation in the microtube and connecting tubing was evaluated using the time-dependent *I*_mc_ and AI. In addition, the vibration speed of the thermal mixer (*Ω*_m_) was varied to determine whether mechanical mixing could suppress RBC sedimentation and improve the stability of AI measurement [90,91].

**Figure 5:**
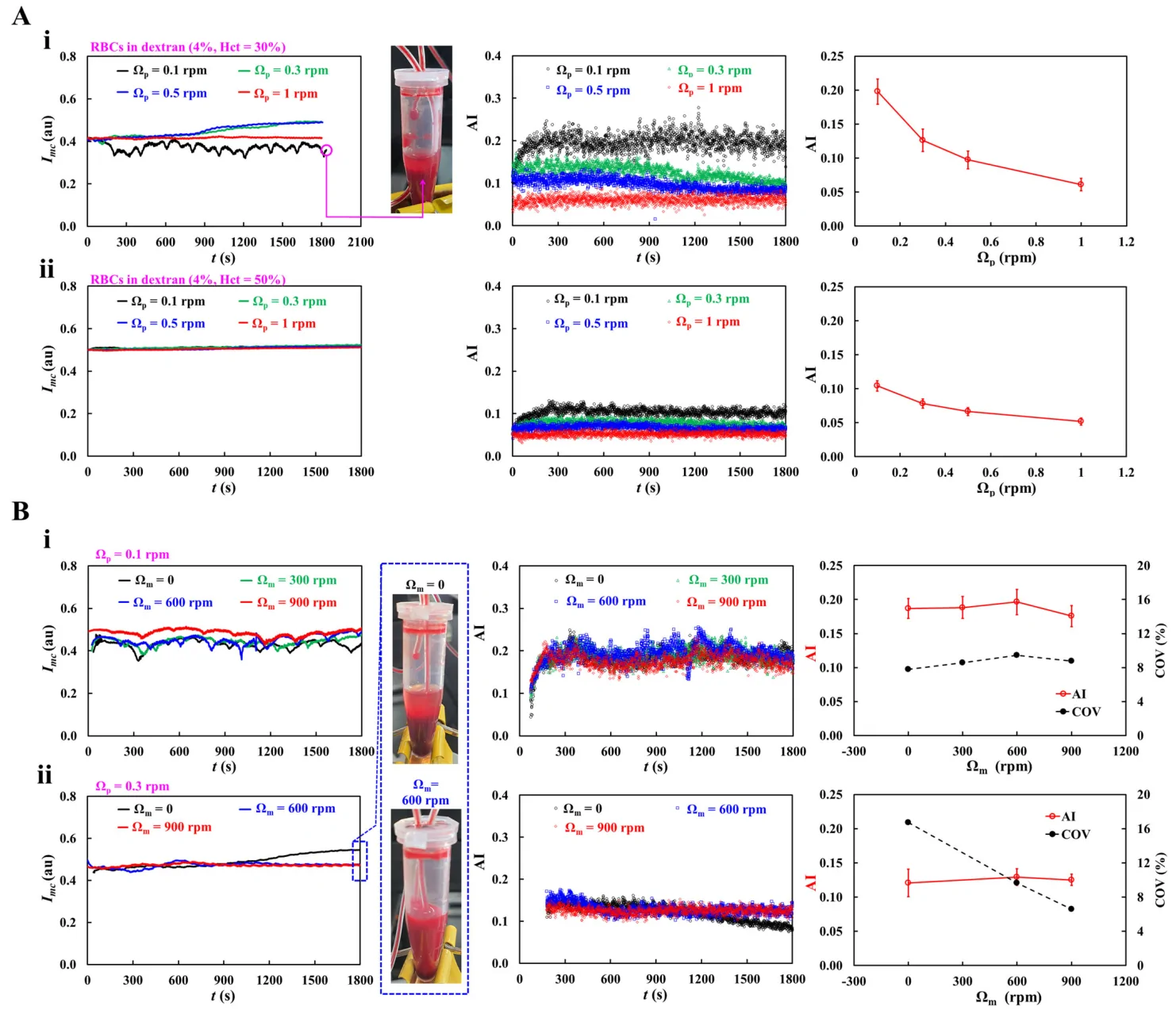
Effect of RBC sedimentation on RBC aggregation index (AI) and suppression of its adverse influence on stable AI measurement. (**A**) Effect of pump speed (*Ω_p_*) on RBC sedimentation and AI. Test blood (Hct = 30% and 50%) was prepared by suspending normal RBCs in 4% dextran solution. Experiments were carried out without mixing. (**i**) Effect of pump speed on AI for test blood (Hct = 30%). The left panel shows time-dependent *I_mc_* at *Ω_p_* = 0.1~1.0 rpm. The snapshot showed RBC sedimentation in the microtube at the end of the experiment at *Ω_p_* = 0.1. The middle panel shows time-lapse AI at different pump speeds. The right panel summarizes variations of AI with respect to *Ω_p_*. (**ii**) Effect of pump speed on AI for test blood (Hct = 50%). The left panel shows time-dependent *I_mc_* as a function of *Ω_p_*. The middle panel shows time-lapse AI at different pump speeds. The right panel exhibits variations of AI with respect to *Ω_p_*. (**B**) Effect of the mixer vibration on suppression of RBC sedimentation and stabilization of AI measurements. (**i**) At *Ω_p_* = 0.1 rpm, the left panel shows time-dependent *I_mc_* at *Ω_m_* = 0, 300, 600, and 900 rpm. The middle panel shows the corresponding time-lapse AI. The right panel summarizes variations of AI and COV as a function of *Ω_m_*. (**ii**) At *Ω_p_* = 0.3 rpm, the left panel shows time-dependent *I_mc_* at *Ω_m_* = 0, 600, and 900 rpm. The snapshots showed clear RBC sedimentation without mixing (*Ω_m_* = 0) and suppression of RBC sedimentation with mixing (*Ω_m_* = 600 rpm) at the end of the experiment. The middle panel shows the corresponding time-lapse AI. The right panel summarizes variations of AI and COV as a function of *Ω_m_*.

As shown in Figure 5A, the effects of pump speed (*Ω_p_*) on RBC sedimentation and AI were evaluated by continuously monitoring *I_mc_* and AI. Because AI was strongly influenced by hematocrit [2,55], test blood was prepared at Hct = 30% and 50% by suspending normal RBCs in 4% dextran solution. To isolate the effect of pump speed, the experiments were conducted without mixer vibration (*Ω*_m_ = 0). For Hct = 30%, as shown in Figure 5A(i), the pump speed (*Ω_p_*) was varied from 0.1 rpm to 1.0 rpm. The left panel showed the time-dependent variation of *I_mc_*. At the lowest pump speed (*Ω_p_* = 0.1 rpm), pronounced RBC sedimentation occurred in the microtube, and a clear RBC-free layer was observed at the end of the experiment. At *Ω*_p_ = 0.3 and 0.5 rpm, *I_mc_* gradually increased over time, indicating a progressive change in the RBC concentration of blood entering the microfluidic chip. In contrast, *I_mc_* remained nearly constant at *Ω_p_* = 1.0 rpm, suggesting more stable blood delivery with reduced influence of RBC sedimentation. The middle panel shows time-dependent changes in AI at different pump speeds. Overall, AI decreased as *Ω_p_* increased. At *Ω_p_* = 0.1 rpm, AI showed large fluctuations over time, mainly due to severe RBC sedimentation and unstable blood delivery. At *Ω_p_* = 0.3 and 0.5 rpm, AI gradually decreased during circulation. In contrast, at *Ω_p_* = 1.0 rpm, AI remained relatively stable with much smaller temporal variations. As represented in the right panel, the corresponding AI value at each pump speed was summarized as AI = 0.198 ± 0.019 at *Ω_p_* = 0.1 rpm (*n* = 1586), AI = 0.126 ± 0.016 at *Ω_p_* = 0.3 rpm (*n* = 1798), AI = 0.097 ± 0.013 at *Ω_p_* = 0.5 rpm (*n* = 1807), and AI = 0.061 ± 0.009 at *Ω_p_* =1.0 rpm (*n* = 1800). These results indicated that low pump speed promoted RBC sedimentation and increased AI fluctuations, whereas higher pump speeds improved the stability of blood delivery and reduced the temporal variation of AI. In contrast, for Hct = 50%, Figure 5A(ii) shows the effect of pump speed on *I_mc_* and AI. As shown in the left panel, *I_mc_* remained nearly constant over time at all tested pump speeds, indicating that RBC sedimentation had little influence on blood delivery at the higher hematocrit. The middle panel shows the corresponding time-dependent AI. Although AI was higher at lower pump speeds, it remained relatively stable over time for each *Ω_p_* condition. As shown in the right panel, AI was plotted against *Ω_p_*. The corresponding AI value at each pump speed was summarized as AI = 0.104 ± 0.008 at *Ω_p_* = 0.1 rpm (*n* = 1553), AI = 0.078 ± 0.007 at *Ω_p_* = 0.3 rpm (*n* = 1608), AI = 0.066 ± 0.006 at *Ω_p_* = 0.5 rpm (*n* = 1801), and AI = 0.052 ± 0.005 at *Ω_p_* =1.0 rpm (*n* = 1845). Compared with Hct = 30%, the Hct = 50% blood showed markedly smaller temporal fluctuations in both *I_mc_* and AI, particularly at low pump speeds. These results indicated that the higher hematocrit reduced the influence of RBC sedimentation during circulation, leading to more stable but lower AI values.

The results showed that AI increased markedly at lower hematocrit and lower pump speeds. However, severe RBC sedimentation also produced large temporal fluctuations. To overcome this limitation, the thermal mixer was operated at different vibration speeds (*Ω*_m_) to suppress RBC sedimentation and improve the stability of AI measurement. Herein, test blood (Hct = 30%) was prepared by suspending normal RBCs in 4% dextran solution. As shown in Figure 5B(i), the effect of thermal-mixer vibration on RBC sedimentation was evaluated at the lowest pump speed (*Ω_p_* = 0.1 rpm) by varying *Ω_m_* from 0 to 900 rpm. The left panel showed that *I_mc_* exhibited large temporal fluctuations at all vibration speeds. Similarly, the corresponding AI values in the middle panel fluctuated markedly and showed no consistent dependence on *Ω_m_*. As summarized in the right panel, the AI remained nearly unchanged with increasing vibration speed, whereas the COV tended to increase at higher *Ω_m_*. From the results, at *Ω_p_* = 0.1 rpm, mixer vibration alone was insufficient to suppress sedimentation-induced instability and did not improve the stability of AI measurement. As shown in Figure 5B(ii), at *Ω_p_* = 0.3 rpm, the effect of thermal-mixer vibration was further evaluated as a function of *Ω_m_* = 0, 600, and 900 rpm. In the left panel, *I_mc_* gradually increased without mixer vibration (*Ω_m_* = 0), consistent with progressive RBC sedimentation. The corresponding snapshot at the end of the experiment clearly showed an RBC-depleted layer in the microtube. In contrast, at *Ω_m_* = 600 and 900 rpm, *I_mc_* remained nearly constant over time, and the snapshot at *Ω_m_* = 600 rpm confirmed that RBC sedimentation was effectively suppressed. The middle panel shows the corresponding time-dependent AI. Without mixer vibration, AI gradually decreased during circulation, whereas it remained relatively stable at *Ω_m_* = 600 and 900 rpm. As summarized in the right panel, the mean AI showed little dependence on *Ω*_m_, while the COV progressively decreased as the vibration speed increased. These results demonstrated that thermal-mixer vibration effectively suppressed RBC sedimentation at *Ω*_p_ = 0.3 rpm and substantially improved the temporal stability and reproducibility of AI measurement.

From the experimental investigation, RBC sedimentation strongly affected AI stability, particularly at low hematocrit and low pump speed, where large temporal fluctuations were observed. At *Ω_p_* = 0.3 rpm, thermal-mixer vibration at *Ω_m_* = 600~900 rpm effectively suppressed RBC sedimentation and maintained stable *I_mc_* and AI. In the following experiments, pump speed and mixer vibration were set to *Ω_p_* = 0.3 rpm and *Ω_m_* = 900 rpm, respectively. These results demonstrated that mixer vibration effectively minimized sedimentation-induced instability, thereby enabling reliable and consistent AI measurement during continuous blood circulation [32,89,90,92,93].

### 3.4. Continuous Measurement of RBC Aggregation at Different Dextran Concentrations in a Closed-Loop Fluidic Circuit

Under the optimized operating conditions (*Ω_m_* = 900 rpm, *Ω_p_* = 0.3 rpm), the closed-loop system was further tested for continuous measurement of different levels of RBC aggregation. To generate a range of aggregation levels, test blood (Hct = 30%) was prepared using dextran solutions with *C_dex_* = 1~4%. The resulting AI values were then evaluated to determine whether the system could provide stable and clearly distinguishable measurements across different dextran concentrations.

**Figure 6:**
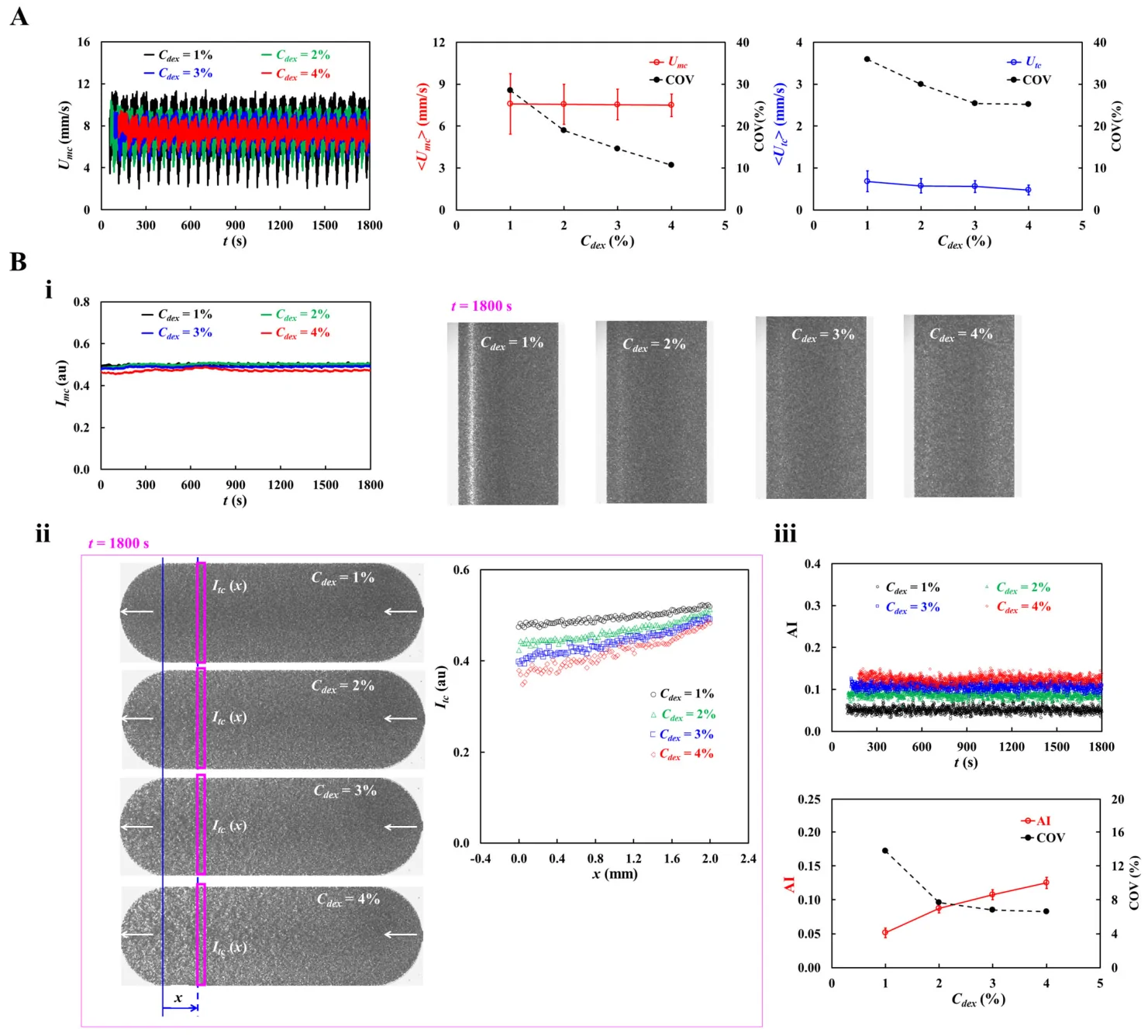
Continuous measurement of RBC aggregation at different dextran concentrations in a closed-loop fluidic circuit. Test blood (Hct = 30%) was prepared by suspending normal RBCs in dextran solutions (*C_dex_* = 1~4%). The pump and mixer were operated at *Ω_p_* = 0.3 rpm and *Ω_m_* = 900 rpm, respectively. (**A**) Effect of dextran concentration on flow stability. The left panel shows time-dependent *U_mc_* at *C_dex_* = 1~4%. The middle and right panels summarize the mean velocities (<*U_mc_*>, <*U_tc_*>) and their COVs as a function of *C_dex_*. (**B**) Effect of dextran concentration on AI. (**i**) The left panel shows time-dependent *I_mc_* as a function of *C_dex_*. In the right panel, representative microscopic images of the main channel acquired at the end of the experiment (*t* = 1800 s) are shown for *C_dex_* =1, 2, 3, and 4%. (**ii**) Spatial distributions of *I_tc_* in the test chamber. The left panel shows representative microscopic images acquired at *t* =1800 s. The right panel shows the corresponding *I_tc_* profiles against flow direction (*x*) with respect to *C_dex_*. (**iii**) The upper panel shows time-dependent AI as a function of *C_dex_*. The lower panel summarizes variations of AI and COV as a function of *C_dex_*.

As shown in Figure 6A, flow stability was evaluated by monitoring the time-dependent *U_mc_* and *U_tc_* at different dextran concentrations (*C_dex_* = 1~4%). The left panel shows that fluctuations in *U_mc_* became progressively smaller as *C_dex_* increased. The middle and right panels summarize the mean velocities (<*U_mc_*>, <*U_tc_*>) and their corresponding COVs. Although the mean velocities remained nearly constant over the tested dextran concentrations, the temporal fluctuations in both *U_mc_* and *U_tc_* were markedly reduced at higher *C_dex_*, resulting in a gradual decrease in COV. Higher dextran concentrations increased fluid viscosity and consequently increased hydraulic resistance. In combination with the compliance of the FS, the increased resistance likely enhanced the damping of pump-induced flow fluctuations, resulting in more stable flow [60,62,78,94]. Overall, the results consistently demonstrated that increasing dextran concentration enhanced flow stability without substantially changing the mean flow velocity. As shown in Figure 6B(i), the time-dependent *I*mc was monitored at *C_dex_* = 1~4%. Representative microscopic images of the main channel acquired at *t* = 1800 s are shown in the right panel. Although the absolute level of *I_mc_* varied with respect to *C_dex_*, the *I_mc_* remained nearly constant throughout the 30 min measurement, indicating stable blood delivery over time. Figure 6B(ii) shows the spatial distribution of *I_tc_* within the test chamber. Representative images acquired at *t* = 1800 s are presented in the left panel, with blood flowing from right to left. As *C_dex_* increased, the image intensity decreased, and the spatial variation of *I_tc_* along the flow direction became more pronounced. The corresponding *I_tc_* profiles in the right panel clearly demonstrate that higher dextran concentrations produced stronger intensity changes within the test chamber, consistent with enhanced RBC aggregation. Finally, Figure 6B(iii) summarizes the time-dependent AI and its variation with respect to *C*_dex_. For all dextran concentrations, AI remained stable over the 30 min circulation period. More importantly, the mean AI increased consistently with increasing *C_dex_*, whereas the corresponding COV gradually decreased. These results demonstrated that the proposed closed-loop system could continuously distinguish different levels of RBC aggregation while maintaining stable and reproducible AI measurements over time.

From the experimental results, under the optimized operating conditions, the closed-loop system provided stable AI measurements for 30 min across *C*_dex_ = 1~4%. The mean AI increased consistently with respect to dextran concentration, demonstrating that the proposed method could clearly distinguish different levels of RBC aggregation, consistent with previous microfluidic studies showing dextran-dependent increases in AI [32,39,67]. Furthermore, the temporal stability of AI supports the feasibility of continuous and reproducible monitoring of RBC aggregation during blood circulation [37,41].

### 3.5. Continuous Monitoring of Temperature-Associated Changes in RBC Aggregation

Because the proposed system enabled stable and continuous measurement of the RBC aggregation index (AI) for at least 30 min, it was further applied to evaluate thermally induced alterations in RBC aggregation under continuous circulation. Thermal exposure occurred in the microtube installed in the thermal mixer, where the temperature was varied from 25 to 47 °C. In contrast, AI was measured in the microfluidic chip maintained at 25 °C during circulation. Accordingly, temporal changes in AI were monitored at a fixed measurement temperature of 25 °C following exposure to different temperatures to assess whether progressive changes in RBC aggregation could be detected in real time fashion.

To demonstrate the capability of the proposed system for detecting thermally induced RBC alterations, test blood (Hct = 30%) was prepared by suspending normal RBCs in a 4% dextran solution. During continuous circulation, to maintain stable blood flow and minimize RBC sedimentation, the peristaltic pump and thermal mixer were operated at *Ω_p_* = 0.3 rpm and *Ω_m_* = 900 rpm, respectively. The circulating blood was exposed to controlled temperatures ranging from *T* = 25 to 47 °C for 30 min. This temperature range was selected to cover physiological and hyperthermic conditions while remaining below temperatures that may cause rapid hemolysis [11,12,13,42]. Changes in RBC aggregation were continuously monitored throughout the thermal exposure to evaluate their time- and temperature-dependent responses.

**Figure 7:**
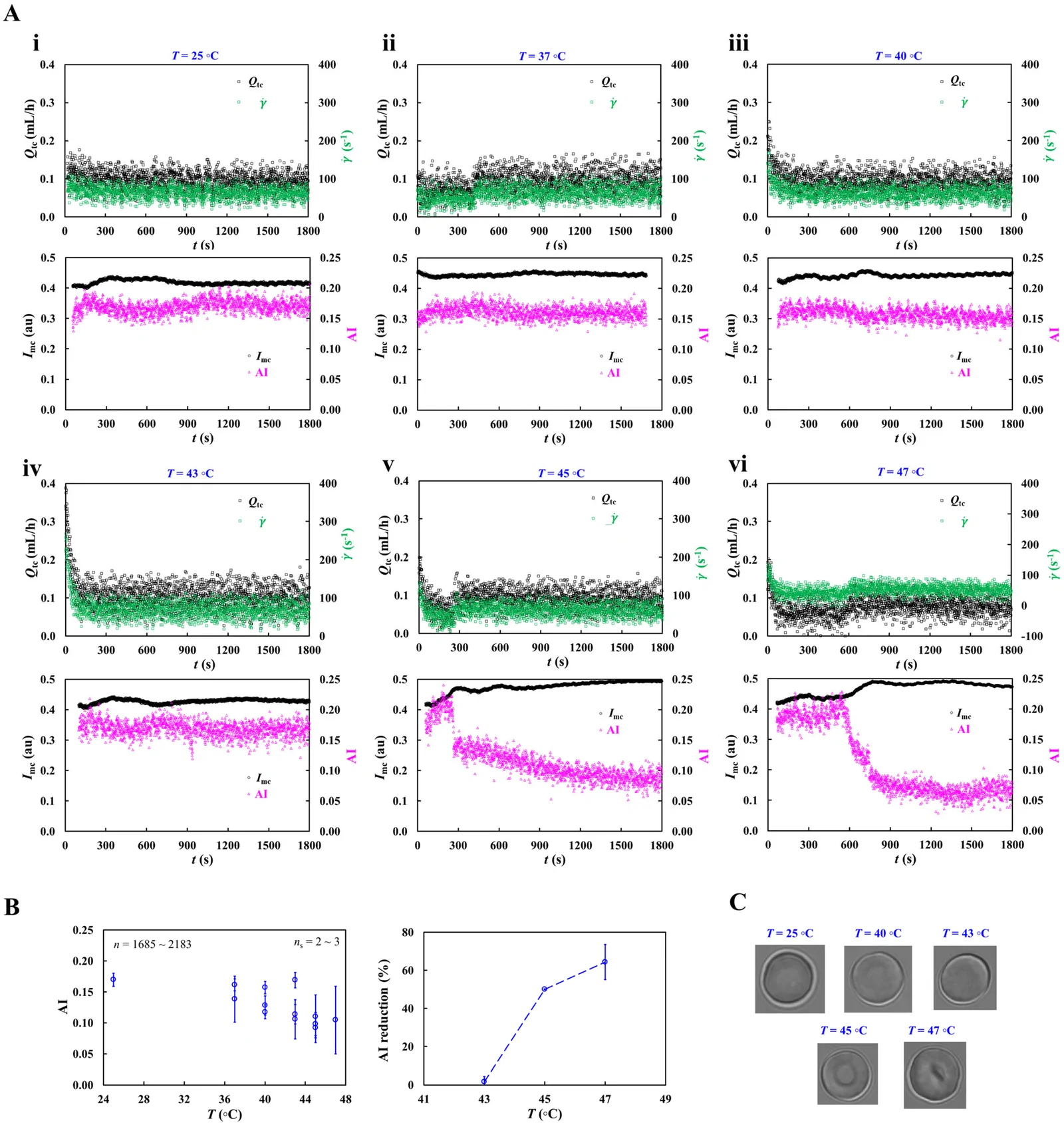
Continuous monitoring of thermally induced changes in RBC aggregation index (AI). Test blood (Hct = 30%) was prepared by suspending normal RBCs in 4% dextran solution. The pump and mixer were operated at *Ω_p_* = 0.3 rpm and *Ω_m_* = 900 rpm, respectively. Using a thermal mixer, the test blood was exposed to temperatures ranging from *T* = 25 to *T* = 47 °C. (**A**) Time-dependent *Q*_tc_, $\dot{\gamma }$, *I_mc_,* and AI with respect to heat-exposure temperature (*T*) ((**i**) *T* = 25 °C, (**ii**) *T* = 37 °C, (**iii**) *T* = 40 °C, (**iv**) *T* = 43 °C, (**v**) *T* = 45 °C, and (**vi**) *T* = 47 °C). (**B**) Variations of AI and AI reduction as a function of thermal exposure temperature. *n*_s_ denotes the number of blood samples tested from a single donor, and *n* denotes the number of temporal AI measurements per experiment. Each point and error bar represent the mean and standard deviation, respectively. (**C**) Microscopic images of RBCs after 30 min of thermal exposure at temperatures ranging from 25 to 47 °C.

As shown in Figure 7A, the test-channel flow rate (*Q*_tc_), estimated shear rate, image intensity (*I*_mc_), and AI were monitored for 30 min at thermal mixer setpoints of 25, 37, 40, 43, 45, and 47 °C. Figure A1B (Appendix A) presents the corresponding main-channel flow rate (*Q*_mc_) and estimated shear rate, which remained relatively stable after the initial transient despite short-term fluctuations. At setpoints up to 43 °C, *I*_mc_ and AI remained approximately stable over time. These observations suggest that prolonged mixing at 900 rpm did not produce an appreciable change in the measured aggregation response over the experimental period. At 45 and 47 °C, however, *I*_mc_ increased while AI decreased markedly. A modest increase in *Q*_tc_ and the test-channel shear rate occurred around the onset of the AI decrease, followed by relatively stable flow conditions. This local shear-rate increase may have contributed to the reduction in AI. Nevertheless, AI continued to decrease after the flow rate and estimated shear rate had approximately stabilized, particularly at 45 °C. The continued AI decrease was not accompanied by a sustained increase in test-channel flow.

Figure 7B summarizes AI and AI reduction measured at 25 °C following thermal exposure at different temperatures. Mean AI was generally lower at exposure temperatures of 45~47 °C than at 25 °C. Two to three blood samples from a single donor were tested with respect to exposure temperature, with 1685~2183 temporal measurements per experiment. Values were presented as mean ± standard deviation within each experiment, reflecting temporal variation rather than between-sample variability. The percentage decrease in AI over the 30 min experiment was calculated as (AI_base_ − AI_final_)/AI_base_ × 100, where AI_base_ and AI_final_ denote the baseline and final AI values, respectively. AI decreased by approximately 2%, 50%, and 65% at thermal mixer setpoints of 43, 45, and 47 °C, respectively. Consistent with the temporal AI profiles, these values indicated a greater overall reduction at higher setpoints. This measure described the net decrease over 30 min, rather than the rate at which AI changed.

To visualize this transition more clearly, at *T* = 45 °C, the lower panel of Figure A1C (Appendix A) presents representative blood-flow images obtained at *t* = 180, 300, 1200, and 1800 s, showing the progressive changes in blood-flow appearance accompanying the reduction in AI. Furthermore, Figure 7C shows representative RBC images after 30 min of thermal exposure. Changes in the central appearance of RBCs are more apparent at 45 and 47 °C than at lower temperatures. These qualitative observations suggest possible morphological alterations but do not independently confirm RBC damage.

The temperature-dependent changes observed at *T* ≥ 45 °C are consistent with previous studies showing that elevated temperatures could substantially alter RBC membrane properties and cellular morphology [11,14]. In particular, thermal exposure above approximately 45 °C has been associated with accelerated hemolysis and pronounced morphological changes [11], while an irreversible increase in RBC membrane stiffness has been reported at approximately 46~50 °C [14]. These heat-induced alterations may modify RBC–RBC interactions and the optical characteristics of flowing blood, thereby contributing to the progressive increase in *I_mc_* and the concurrent decrease in AI observed in the present study. The *I*_mc_ represented the mean grayscale intensity within the selected region of the main channel. An increase in *I*_mc_ may reflect changes in local RBC concentration, cell shape or orientation, or light transmission and scattering. Because these contributions could not be separated in the present study, the observed increase in *I*_mc_ was interpreted as a temperature-associated optical change, not as direct evidence of RBC damage. In addition, RBC deformability and aggregation are important determinants of blood rheology and microcirculatory flow. Deterioration of RBC mechanical properties could increase flow resistance and disturb normal cell transport through the microcirculation [2].

Overall, the contrasting responses of *I*_mc_ and AI at *T* ≥ 45 °C indicated the beginning of progressive heat-related impairment in RBC hemorheological properties. Notably, the substantial decrease in AI highlighted the potential of the proposed system as a sensitive, real-time tool for detecting thermally induced changes in circulating RBCs.

In this demonstration, the 4% dextran solution was selected to enhance baseline RBC aggregation and facilitate detection of AI decreases during thermal exposure. However, dextran affects both aggregation and suspending-medium viscosity. Temperature-related changes in suspension viscosity may therefore alter flow resistance and local shear conditions. As shown in Figure 7A, the test-channel flow rate and estimated shear rate increased modestly around the onset of the AI decrease at thermal mixer setpoints of 45 and 47 °C. These flow changes may have contributed to the observed AI decrease, although their cause was not established. Because suspending-medium viscosity was not independently measured during thermal exposure, the effects of medium viscosity, local flow conditions, and RBC aggregation changes could not be fully separated.

The use of RBCs suspended in dextran rather than native whole blood limits the physiological interpretation of the present results. Although previous studies reported correlated aggregation tendencies in dextran and plasma [95] and slower aggregation after RBC heat treatment in both media [16,21], these findings do not establish equivalent thermal responses. Oxygenation and RBC membrane properties can also influence temperature-dependent aggregation [96]. Therefore, the observed AI changes should be interpreted within the tested suspension and experimental conditions. Future comparisons with whole blood under matched hematocrit, oxygenation, thermal exposure, and flow conditions are needed to establish physiological relevance.

The 30 min measurement period does not represent an established upper limit of platform operation. At the measured mean test-channel flow rate, continuous discharge would remove approximately 80 µL over 1 h, equivalent to 8% of the initial sample volume. Returning the outflow from port (c) to the microtube could reduce sample loss during longer experiments; however, the resulting changes in hydraulic resistance would require reassessment of the flow distribution and baseline AI. Extended operation would also require verification of flow and temperature stability, sample retention, and potential baseline drift associated with prolonged circulation and mixing. Measurements beyond 1 h remain to be experimentally validated.

Several limitations should be considered when interpreting these findings. First, temperature-associated changes in RBC aggregation were assessed using AI, but their relationship to RBC injury was not independently established through measurements of hemolysis, deformability, membrane stability, or morphology [11,97,98]. Second, the use of RBC suspensions in dextran solution does not fully reproduce the hemorheological environment of whole blood, because RBC aggregation and thermal responses can be strongly influenced by the composition of the suspending medium, hematocrit, and plasma proteins [84,97,99]. Future studies combining whole-blood experiments with simultaneous biochemical, morphological, and mechanical measurements will therefore be important for establishing the mechanistic basis of AI changes and validating AI as a reliable marker of thermally induced RBC damage. Third, blood temperature was not directly measured during blood circulation. The reported temperatures were the thermal mixer settings, not the actual blood temperatures. Future measurements will be needed to confirm whether the blood reaches the set temperature and identify the blood temperatures associated with the observed AI changes. At last, microfluidic measurements were performed under ambient conditions of 25 °C rather than at physiological temperature. Future work will incorporate temperature control at the microfluidic measurement site to enable measurements at 37 °C [11,97,98]. Second, the use of RBC suspensions in dextran solution does not fully reproduce the hemorheological environment of whole blood, because RBC aggregation and thermal responses can be strongly influenced by the composition of the suspending medium, hematocrit, and plasma proteins [84,97,99]. Future studies combining whole-blood experiments with simultaneous biochemical, morphological, and mechanical measurements will therefore be important for establishing the mechanistic basis of AI changes and validating AI as a reliable marker of thermally induced RBC damage. Third, blood temperature was not directly measured during blood circulation. The reported temperatures were the thermal mixer settings, not the actual blood temperatures. Future measurements will be needed to confirm whether the blood reaches the set temperature and identify the blood temperatures associated with the observed AI changes. Finally, microfluidic measurements were performed under ambient conditions of 25 °C rather than at physiological temperature. Future work will incorporate temperature control at the microfluidic measurement site to enable measurements at 37 °C.

## 4. Conclusions

In this study, a closed-loop microfluidic platform was proposed for continuous monitoring of RBC aggregation during prolonged blood circulation and thermal exposure. The system integrated a peristaltic pump, a flow stabilizer (FS), a microfluidic chip, and a thermal mixer, which enabled stable circulation, suppression of RBC sedimentation, controlled thermal exposure, and real-time measurement of the aggregation index (AI). In particular, the FS markedly suppressed pump-induced flow fluctuations by increasing the characteristic flow-response time by approximately 17.6-fold, thereby substantially reducing pulsatile flow generated in a closed-loop fluidic circuit.

The optimized operating conditions of pump speed (*Ω_p_* = 0.3 rpm) and mixer-vibration (*Ω_m_* = 900 rpm) further enabled continuous AI measurement for at least 30 min, while minimizing RBC sedimentation and maintaining distinct shear environments for RBC disaggregation in the main channel and aggregation in the test chamber. The system successfully detected dextran-dependent variations in RBC aggregation under continuous circulation, demonstrating its capability to resolve changes in hemorheological behavior without repeated flow cessation or blood sampling. Most importantly, during controlled thermal exposure, *I_mc_* and AI remained nearly unchanged at temperatures up to 43 °C, whereas a clear transition occurred at ≥ 45 °C, where *I_mc_* progressively increased and AI markedly decreased with respect to exposure time. These opposing responses revealed the onset and progression of heat-induced deterioration in RBC hemorheological properties and demonstrated that AI could serve as a sensitive functional indicator of thermally induced RBC alterations.

Overall, the proposed platform provides a distinct advantage over conventional end-point measurements by enabling repeated assessment of the same circulating RBC population under continuously controlled flow and thermal conditions. With further validation against established biochemical, morphological, and mechanical markers of RBC damage, this approach could provide a versatile tool for studying thermal injury, blood-storage lesions [100], mechanical fatigue, and other progressive changes in RBC function under physiologically relevant circulation conditions.

## Figures and Tables

**Table 1:** Comparison of the present platform with the authors’ previous microfluidic platforms for assessing RBC aggregation.

Ref.	Closed-Loop Operation	Flow Stabilization	Thermal Exposure	Sedimentation Suppression Management	AI Measurement	Continuous AI Monitoring During Thermal Exposure
[37]	Yes (Peristatic pump)	Air compliance	No	No	Periodic AI	No
[38]	Yes (Peristaltic pump)	Air compliance	No	No	Continuous AI	No
[39]	No (Syringe pump)	No	No	Sedimentation effect examined	Continuous AI	No
[33]	No (Syringe pump)	No	Pretreatment before experiment	High flow rate during blood viscosity measurement	Continuous AI	No
[41]	No (Syringe pump)	No	No	High flow rate during blood viscosity measurement	Continuous AI	No
[40]	No (Syringe pump)	No	No	No	Continuous AI	No
Present study	Yes (Peristatic pump)	Microfluidic stabilizer	Thermal mixer Integrated	Mixer vibration	Continuous AI	Yes

## Data Availability

The original contributions presented in this study are included in the article. Further inquiries can be directed to the corresponding author.

## Appendix A

### Appendix A.3

**Table A1:** Effect of pulsation period (*T*_p_) on main-channel blood velocity (*U*_mc_) with and without the flow stabilizer (FS).

*T*_p_ (s)	Without FS			With FS			Percentage Reduction in COV (%)
	Mean	Standard Deviation	COV (%)	Mean	Standard Deviation	COV (%)	
10	18.72	5.66	30.26	17.62	0.76	4.30	85.77
20	18.51	7.87	42.52	17.40	1.85	10.65	74.96
40	18.03	8.21	45.51	17.99	3.54	19.67	56.78
60	17.99	8.19	45.50	17.18	4.54	26.44	41.90
80	18.00	8.19	45.50	18.09	5.48	30.32	33.37
100	18.34	8.23	44.84	17.69	5.83	32.94	26.50
